# Volatiles and Refractories in Surface-Bounded Exospheres in the Inner Solar System

**DOI:** 10.1007/s11214-021-00833-8

**Published:** 2021-06-16

**Authors:** Cesare Grava, Rosemary M. Killen, Mehdi Benna, Alexey A. Berezhnoy, Jasper S. Halekas, François Leblanc, Masaki N. Nishino, Christina Plainaki, Jim M. Raines, Menelaos Sarantos, Benjamin D. Teolis, Orenthal J. Tucker, Ronald J. Vervack, Audrey Vorburger

**Affiliations:** 1 Southwest Research Institute, San Antonio, TX USA; 2 NASA Goddard Space Flight Center, Greenbelt, MD USA; 3 University of Maryland Baltimore County, Baltimore, MD USA; 4 Sternberg Astronomical Institute, Moscow State University, Moscow, Russia; 5 Institute of Physics, Kazan Federal University, Kazan, Russia; 6 Department of Physics and Astronomy, University of Iowa, Iowa City, IA USA; 7 LATMOS/CNRS, Sorbonne Université, UVSQ, IPSL, Paris, France; 8 Institute of Space and Astronautical Science, Japan Aerospace Exploration Agency, Sagamihara, Kanagawa Japan; 9 Italian Space Agency, Rome, Italy; 10 Department of Climate and Space Sciences and Engineering, University of Michigan, Ann Arbor, MI USA; 11 Johns Hopkins Applied Physics Laboratory, Laurel, MD USA; 12 Physikalisches Institut, University of Bern, Bern, Switzerland

**Keywords:** Moon, Mercury, Exosphere, Refractories, Volatiles, Solar wind, Magnetosphere, Neutrals, Ions

## Abstract

Volatiles and refractories represent the two end-members in the volatility range of species in any surface-bounded exosphere. Volatiles include elements that do not interact strongly with the surface, such as neon (detected on the Moon) and helium (detected both on the Moon and at Mercury), but also argon, a noble gas (detected on the Moon) that surprisingly adsorbs at the cold lunar nighttime surface. Refractories include species such as calcium, magnesium, iron, and aluminum, all of which have very strong bonds with the lunar surface and thus need energetic processes to be ejected into the exosphere. Here we focus on the properties of species that have been detected in the exospheres of inner Solar System bodies, specifically the Moon and Mercury, and how they provide important information to understand source and loss processes of these exospheres, as well as their dependence on variations in external drivers.

## Introduction

Volatiles and refractories are subject to different loss and source processes, and each provides different insights on the behavior of the exospheres of such species. Calcium and magnesium, for example, are predominantly ejected via micrometeoroid impact vaporization (probably in molecular compounds) and (to a lesser extent) sputtering; therefore, they are species of interest to study the exospheric response to micrometeoroid flux (Janches et al. 2021). On the other hand, helium is an element of predominantly solar wind origin that has been detected at both Mercury and the Moon. As such, it offers the opportunity to study the response to the same external driver (solar wind flux) of two very different exospheres: one (Mercury’s) embedded in its own magnetosphere; the other (the Moon’s) directly exposed to solar wind bombardment except for ∼1/6 of its orbit when the solar wind is effectively shielded by the Earth’s magnetotail. In this regard, it is fortunate that the two most prominent surface-bounded exospheres in the inner Solar System for which we have measurements are so different, as they highlight the relative importance of different source and loss processes. We discuss volatiles and refractories in Sects. 2 and 3, respectively. Section 4 discusses the “missing” species, i.e. those for which a detection has been expected in these exospheres but so far have not been achieved. Section 5 briefly discusses ions and Energetic Neutral Atoms, as they also play an important role in determining the loss rate and composition of a surface-bounded exosphere. Section 6 recaps the overall discussion. Future considerations for needed laboratory measurements, modeling improvements, and further observations are summarized in Sect. 7. Species with different volatility, such as the alkalis Na and K and OH/H_2_O, are discussed in Leblanc et al. (2021) and Schörghofer et al. (2021), respectively.

## Volatiles

This section discusses the species with the highest volatility (and hence mobility), including the two most prominent noble gases, helium (Sect. 2.1) and argon (Sect. 2.2). These are the species for which a solid database of observations exists (for helium at both Mercury and the Moon), and they represent endogenic species (^40^Ar much more than ^4^He). Argon, in particular, is important in studying how surface-bounded exospheres are shaped by temporary cold trapping. Section 2.3 closes with a discussion of other volatiles, most of which give insights into how the exosphere reacts to the variations in the solar wind.

### Helium

Helium (^4^He) has been detected on both the Moon and Mercury. In both cases, the dominant source of exospheric helium is implantation of solar wind alpha particles (He^++^) on the surface and their subsequent release into the exosphere as neutrals.

**Fig. 1:**
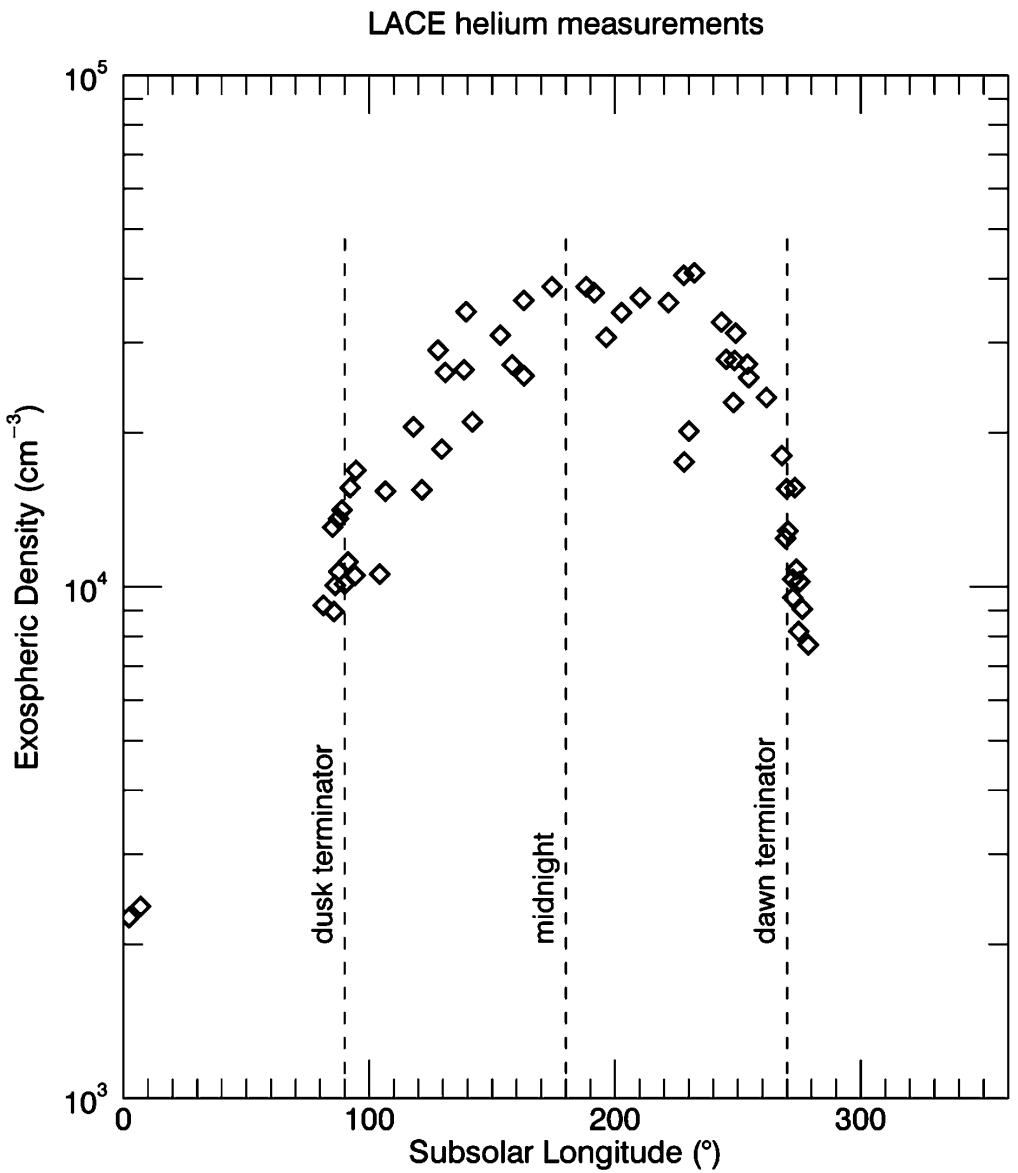
Exospheric number densities for ^4^He measured at the lunar surface by the LACE mass spectrometer (Apollo 17) during nine lunations in 1972 and 1973. Subsolar longitudes are angles from the subsolar point. The two points at noon represent sporadic checks when the instrument was briefly turned on at noon. Adapted from Hoffman et al. (1973)

On the Moon, helium was one of the first exospheric species discovered by the Lunar Atmosphere Composition Experiment (LACE) mass spectrometer deployed during the Apollo 17 mission (Hoffman et al. 1973). The measurements, taken during nine lunations at nighttime (during the day, LACE counts were overwhelmed by outgassing from the instrument itself), showed an increase of exospheric surface density from dusk up to ∼2 AM local time (peak of ∼3 × 10^4^ cm^−3^), followed by a decrease towards dawn (see Fig. 1).

This profile was predicted by Hodges and Johnson (1968) and explained as a result of helium atoms not adsorbing even at the cold lunar nighttime surface. As a result, the exospheric density, $n$, is inversely proportional to the surface temperature $T$: $n \sim T^{-5/2}$ (Hodges and Johnson 1968). Correlation between the helium exospheric density measured by LACE and the geomagnetic index (a proxy for solar activity) revealed that alpha particles from the solar wind are the main source of lunar ^4^He (Hodges and Hoffman 1974). These particles continuously bombard the lunar surface unimpeded by a magnetosphere except for when the Moon is inside the Earth’s magnetotail (during ∼2 days around full moon), become neutralized, and finally are released as neutrals into the exosphere. LACE observations were adequately described by an exospheric model in which helium atoms are in thermal equilibrium with the lunar surface and where gravitational escape is the dominant loss process, with photoionization being a secondary but non-negligible loss process (e.g. Hodges 1973).

On Mercury, helium was detected by the UltraViolet Spectrometer (UVS) aboard Mariner 10 (Broadfoot et al. 1974) through observation of the 58.4 nm resonant scattering emission line (HeI). The vertical column density above the subsolar point was $7 \times 10^{11}$ cm^−2^ for a derived subsolar exospheric surface density of $4.5 \times 10^{3}$ cm^−3^. The altitude profile observed above the subsolar point could be explained by a relatively simple exospheric model that assumes complete saturation of Mercury’s surface with helium and a full thermal accommodation with the surface. However, observations taken close to the terminator could not be explained by the same model (Broadfoot et al. 1976).

The fact that at the Moon helium could be reasonably explained by a full thermal accommodation with the surface, whereas at Mercury this appeared not to be the case, was interpreted to originate from the poor knowledge of the gas-surface interaction. The exchange of energy between exospheric atoms and an airless body’s surface is described by the accommodation coefficient $\alpha $ (e.g. Hunten et al. 1988):

$$ \alpha = \frac{E_{\mathit{out}} - E_{\mathit{in}}}{E_{T} - E_{\mathit{in}}} $$

where $E_{\mathit{out}}$ is the energy of the atom or molecule after the collision, $E_{\mathit{in}}$ is its energy prior to the collision, and E_T_ is the energy of the atom in thermal equilibrium with the surface. When $\alpha = 1.0$, $E_{\mathit{out}} = E_{T}$ and the atom leaves the surface with an energy corresponding to thermal equilibrium with the surface. In this case, the surface temperature is what controls the energy of the atoms, and therefore the structure (and escape) of the exosphere. Larger hop length on hotter surfaces implies that non-adsorbable species will accumulate in the nightside exosphere. Conversely, with $\alpha < 1.0$ the exosphere is less dependent on the surface temperature. Early modelers of the lunar exospheres (Hartle and Thomas 1974; Hodges 1975) used $\alpha = 1.0$ on the assumption that the lunar surface is saturated with helium, an assumption based on results from the Apollo 11 Solar Wind Composition experiment (Bühler et al. 1969), which measured the solar wind flux impacting the Moon. This experiment revealed that this flux was high enough to establish saturation within just tens of thousands of years (Banks et al. 1970). When the Mariner 10 observations were published, Hartle et al. (1975) proposed that the mismatch between model and observations at terminator could be caused by not knowing the surface temperature close to the terminator with sufficient accuracy, perhaps owing to shadows cast by nearby reliefs (micro-shadows cast by grains, or macro-shadows cast by ridges and crater rims): if $\alpha = 1.0$ and the surface temperature (and thus $E_{T}$) is not known accurately, then $E_{\mathit{out}}$ is poorly constrained. This would also explain why the altitude profiles above the subsolar point, where the temperature was better constrained, were better explained by the model. However, Shemansky and Broadfoot (1977) and Smith et al. (1978) noted that the atom-surface interaction involves single phonon collisions rather than multiple ones, and that $\alpha $ depends on the Debye characteristic temperature of the surface lattice. Therefore, they postulated that full thermal accommodation was not justified. As such, helium is an important species for studying the gas-surface interaction in exospheres of airless bodies.

Helium is lost primarily via thermal escape. Simulations of the lunar exospheric helium by Hodges (1977a, 1978) that included solar radiation pressure and the gravitational attraction of the Sun and the Earth (besides that of the Moon) supported the existence of a vast helium corona around the Moon. This corona may extend to tens of lunar radii and is populated by satellite helium atoms whose periapsis is higher than the highest peak on the Moon; hence, they spend their entire lifetime in orbit until they are photoionized (after ∼6 months). Some of these atoms may even reach the Earth’s exosphere, suggesting the possibility of the existence of a “shared exosphere” between the Moon and the Earth.

Up to 10% of the lunar helium measured by LACE is not accounted for by the solar wind (Hodges 1975). Hodges (1977b) proposed that this is endogenic lunar helium, coming from the radioactive decay of thorium and uranium within the lunar mantle and crust (Kockarts 1973) and finding its way to the exosphere via cracks or fissures (Killen 2002), the same way ^40^Ar does (see Sect. 2.2). The outgassing rate of endogenic ^4^He would then constrain the amount of radioactive elements in the lunar crust. The challenge is how to distinguish it from the dominant background, i.e., the solar-wind-derived helium. This intriguing topic has been addressed by spacecraft that detected helium in recent years. The Lyman Alpha Mapping Project (LAMP; Gladstone et al. 2010a) far-ultraviolet (FUV) imaging spectrograph onboard the Lunar Reconnaissance Orbiter (LRO; Chin et al. 2007) made the first spectroscopic detection of helium, by observing the HeI emission line at 58.4 nm (Stern et al. 2012). The retrieved surface densities (obtained around dusk local time) were somewhat lower than those from LACE. Subsequent observations confirmed the 4.5-day decay constant (Feldman et al. 2012). In particular, the helium density was observed to decrease as soon as the Moon entered the Earth’s magnetotail, and was thus shielded from the solar wind bombardment. Helium was measured in situ again by the Neutral Mass Spectrometer (NMS; Mahaffy et al. 2014) onboard the Lunar Atmosphere and Dust Environment Explorer (LADEE; Elphic et al. 2014). During LADEE’s 7-month mission, NMS measured helium atom densities at a few tens of km altitude around the equator (Benna et al. 2015; see also Fig. 7). At the same time, the twin spacecraft ARTEMIS (Acceleration, Reconnection, Turbulence and Electrodynamics of the Moon’s Interaction with the Sun; Angelopoulos 2011) was measuring the flux of solar wind alpha particles around the lunar environment. Therefore, Benna et al. (2015) could make a direct comparison between the direct source (solar wind alpha particles) and the resulting neutrals (helium atoms, measured by NMS), and found a positive correlation between the two. They also derived a value for the helium source rate that is not accounted for by the solar wind alpha particles and interpreted it to be the endogenic population mentioned by Hodges (1977b): (1.5-2.0) × 10^6^ cm^−2^ s^−1^, or about 15-20% of the solar wind alpha particles influx, slightly higher than Hodges’ estimate. Benna et al. (2015) also found a 4.5-day escape time constant for lunar exospheric helium, confirming that thermal escape is the major loss process for this exospheric species.

**Fig. 2:**
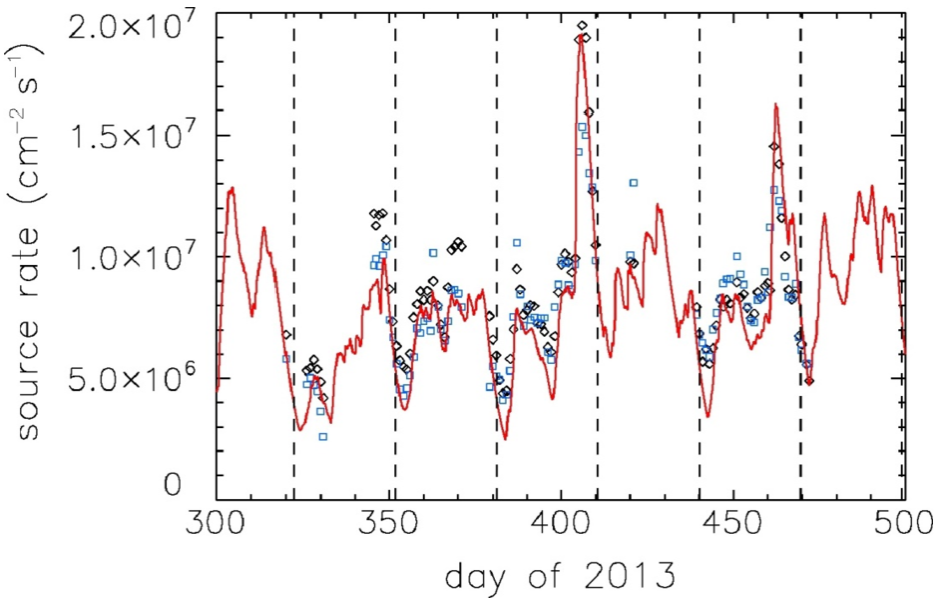
Three different datasets (neutral helium measured in situ by LADEE/NMS: black diamonds; neutral helium measured remotely by LRO/LAMP: blue squares; solar wind alpha particles measured in situ by ARTEMIS/ESA: red line) show strongly correlated source rates between solar wind alpha particles and lunar exospheric helium. Vertical lines indicate times of full moon, when the geomagnetic tail effectively shields the Moon from the solar wind. Reproduced from Hurley et al. (2016)

Later, the same two datasets were compared by Hurley et al. (2016) with LAMP surface densities derived from the HeI emission line. The three datasets, which offered three different “views” of the lunar helium (in situ measurements of neutral atoms and solar wind alpha particles, and remote sensing measurements of neutral atoms), agreed well with each other (see Fig. 2). The derived endogenic source rate, however, was considerably higher than previous estimates and consistent with the one derived by Grava et al. (2016) using targeted LRO off-nadir observations with LAMP: 35-40% of the solar wind. Clearly more observations are needed to constrain this important source rate.

Recently, LAMP carried out a more extensive atmospheric campaign to map the lunar helium over several latitudes, longitudes, and local times, comparing the column densities with ARTEMIS solar wind alpha particles. The result of this multi-year long campaign, with more than 170 orbits, points to an endogenic source rate of 1.49 ± 0.08 × 10^6^ cm^−2^ s^−1^, or about 19% of the solar wind (Grava et al. 2021), in agreement with the LADEE/NMS measurements and slightly higher than the estimates of Hodges (1977b) based on the amount of thorium and uranium within the crust estimated by Taylor and Jakeš (1974) and on the assumption that the outgassing rate is the same as that for ^40^Ar (6% of the total production). The discrepancy might mean that this assumption is wrong (helium is more volatile so its outgassing rate might be higher) or that the outgassing of helium is sporadic, like that of ^40^Ar. Grava et al. (2021) also found that the same dataset can be adequately reproduced by an exospheric model that assumes full thermal accommodation ($\alpha = 1.0$).

Finally, the mass spectrometer CHACE (CHandra’s Altitudinal Composition Explorer; Sridharan et al. 2010) onboard the Moon Impact Probe (MIP) of the Chandrayaan-1 spacecraft (Goswami and Annadurai 2009) attempted the first measurement from a spacecraft of the lunar helium dayside exosphere, but was able to place only an upper limit of 800 cm^−3^ (Das et al. 2017). This low value arises from the combination of several factors: the observations were on the dayside (where the surface density is lowest), obtained during the magnetotail passage of the Moon (when the solar wind — the main source of helium — is deflected by Earth’s magnetosphere and thus has no access to the lunar surface), and close in time to the minimum solar wind flux of cycle 24.

An isotope of helium of great interest is ^3^He, a potential clean energy source. Being scarce in the Earth’s atmosphere and mantle yet abundant on the Moon, where it is delivered by the solar wind, it has gained attention particularly in recent times thanks to the renewed interest in lunar exploration. Thus far the only measurements are those from the surface. The ^3^He content in returned lunar samples correlates well with TiO_2_ content and maturity index Is/FeO (Jordan 1989). Taking into account the estimated solar wind flux on the Moon, the correlation coefficient between the measured ^3^He content and the TiO_2_ content, the solar wind flux, and the maturity parameter in the nine Apollo soil samples studied is 0.944 (Johnson et al. 1999). A similar value, 0.938, was found in 25 Apollo soils by Fa and Jin (2007). These authors estimated the ^3^He content on the surface of the Moon as $C$(^3^He) = 0.56 * $S$(TiO_2_) * ($F$/*OMAT*) + 1.62, where $C$(^3^He) is in ppb, $S$(TiO_2_) is the TiO_2_ content in wt%, $F$ is the normalized solar flux, and *OMAT* is the maturity index taken from Lucey et al. (2000).

A physically plausible model of the observed correlation between ^3^He content, TiO_2_ content, solar wind flux, and soil maturity in returned lunar samples was developed by Shkuratov et al. (1999). In the returned lunar samples, ^3^He and ^4^He are stable at least at room temperature, meaning that these isotopes are strongly bounded in the regolith and have a high activation energy of diffusion in the soil. ^3^He and ^4^He are mainly delivered to the regolith by the solar wind, so that the content of these isotopes on the surface of the Moon should be correlated with the solar wind flux. The ^3^He atoms implanted into the regolith by the solar wind are captured in traps located in vacancies of the crystal grid. This means that the ^3^He content in the soil increases with increasing concentration of such traps. The degree of damage of the crystal lattice (soil maturity) increases with exposure to the solar wind bombardment, and thus with increasing age of the samples. The concentration of ^3^He traps depends on the soil maturity and on the volume fraction of minerals with a high content of vacancies (Scherzer 1983). Experimental works show that irradiation of ilmenite (FeTiO_3_, the main carrier of Ti on the surface of the Moon) by solar wind particles leads to the appearance of radiation-induced defects in the lattice, which are able to trap solar wind ions (Scherzer 1983). Ilmenite is considered to be the most effective He trapper among main lunar minerals because it has a high concentration of vacancies. Incidentally, OH/H_2_O content on the surface of the Moon is also correlated with TiO_2_ content (Wöhler et al. 2017), providing additional evidence that the FeTiO_3_ content is the main factor controlling the behavior of many volatiles on the surface of the Moon.

Maps of the ^3^He content on the lunar surface were calculated using the strong correlations between the ^3^He content and normalized solar wind flux at the point of collection of lunar samples, the TiO_2_ content, and optical maturity in returned lunar samples. Maps from different authors are similar (Johnson et al. 1999; Fa and Jin 2007; Kim et al. 2019). In general, the ^3^He content is higher in the maria than in the highlands. The ^3^He content in low-Ti maria such as Mare Frigoris, Mare Imbrium, and Mare Serenitatis is also low, consistent with the TiO_2_-^3^He relationship mentioned earlier. A moderately high ^3^He content of 10–15 ppb is predicted in Oceanus Procellarum, the Apollo basin, Mare Orientale, Mare Fecunditatis, Mare Crisium, Mare Moscoviense, and Mare Marginis (Kim et al. 2019). The highest ^3^He concentrations of up to about 24 ppb are predicted for Ti-rich parts of Oceanus Procellarum, Mare Fecundidatis, Mare Tranquillitatis, Mare Crisium, Mare Marginis, and Mare Moscoviense (Kim et al. 2019). Hence, the expected ^3^He content on the Moon is highest in the western maria. One could therefore expect an enhancement in exospheric helium there. However, no such enhancement could be detected by either LRO/LAMP (Grava et al. 2021) or LADEE/NMS (Benna et al. 2015). It is noteworthy that LADEE/NMS did detect an enhancement in argon, another endogenic element, in the same region (western maria). A “helium bulge” would be difficult to detect from a single spacecraft, owing to the randomness of the outgassing location and owing to the large scale height and hop length of helium atoms. LAMP is not able to distinguish between ^3^He and ^4^He, and LADEE/NMS did not detect ^3^He. However, a mass spectrometer such as LEMS (Benna et al. 2020), deployed at the lunar surface, would be able to distinguish between the two helium isotopes.

### Argon

**Fig. 3:**
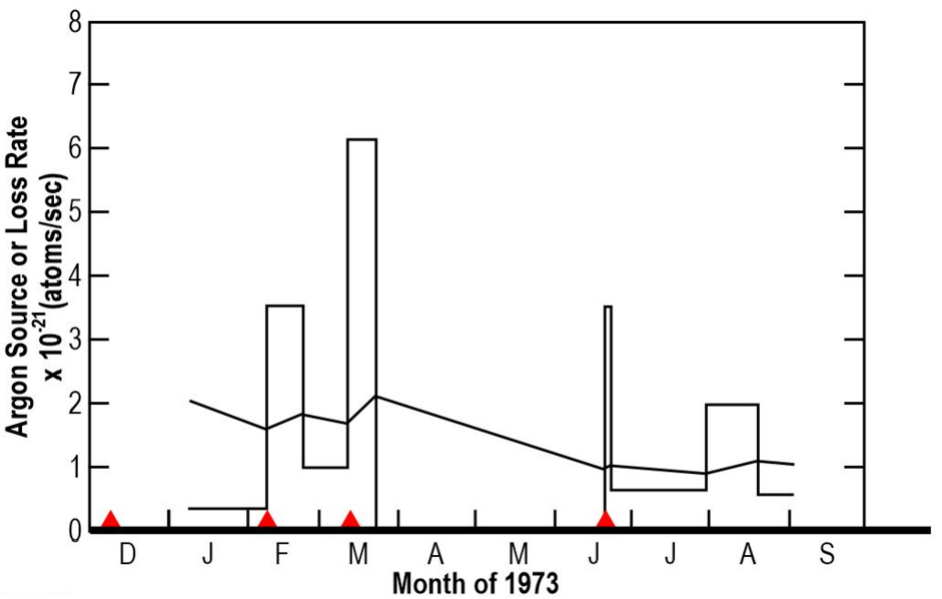
Peaks in exospheric source rate of ^40^Ar measured by LACE (histogram) occurred soon after moonquakes recorded by the Apollo seismometers (red triangles). The black line is the argon exospheric loss rate. Adapted from Hodges (1977b)

Argon (^40^Ar), like helium was discovered by LACE during the Apollo 17 mission. As opposed to the most common isotope, ^36^Ar, which comes from the solar wind, ^40^Ar is an endogenic species, a byproduct of the radiogenic decay of ^40^K within the lunar crust, which is released into the exosphere following diffusion, melting by impacts, or grinding of rocks (Killen 2002). In fact, spikes in lunar argon-40 density measured by LACE occurred soon after high-frequency teleseismic events, or shallow moonquakes, recorded by the Apollo seismometers (Nakamura 1977; Hodges 1977b; see also Fig. 3).

Shallow moonquakes, which probably occur a few tens of km below the surface (Hodges 1981; Killen 2002), may perturb the upper crust allowing the pockets of gas trapped in voids to diffuse out into the exosphere.

**Fig. 4:**
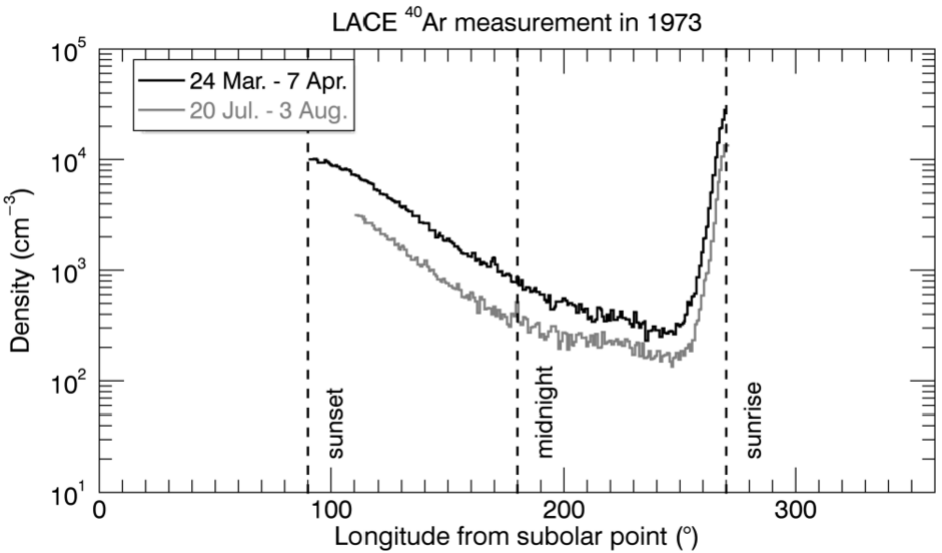
The diurnal profiles obtained four months apart (four lunations) by LACE in 1973. Measurements were made from dusk (90° subsolar longitude) to dawn (270° subsolar longitude). Adapted from Hodges (1975)

The diurnal profile of ^40^Ar resembles that of a species that condenses at the cold nighttime surface and is then released at dawn (Fig. 4).

**Fig. 5:**
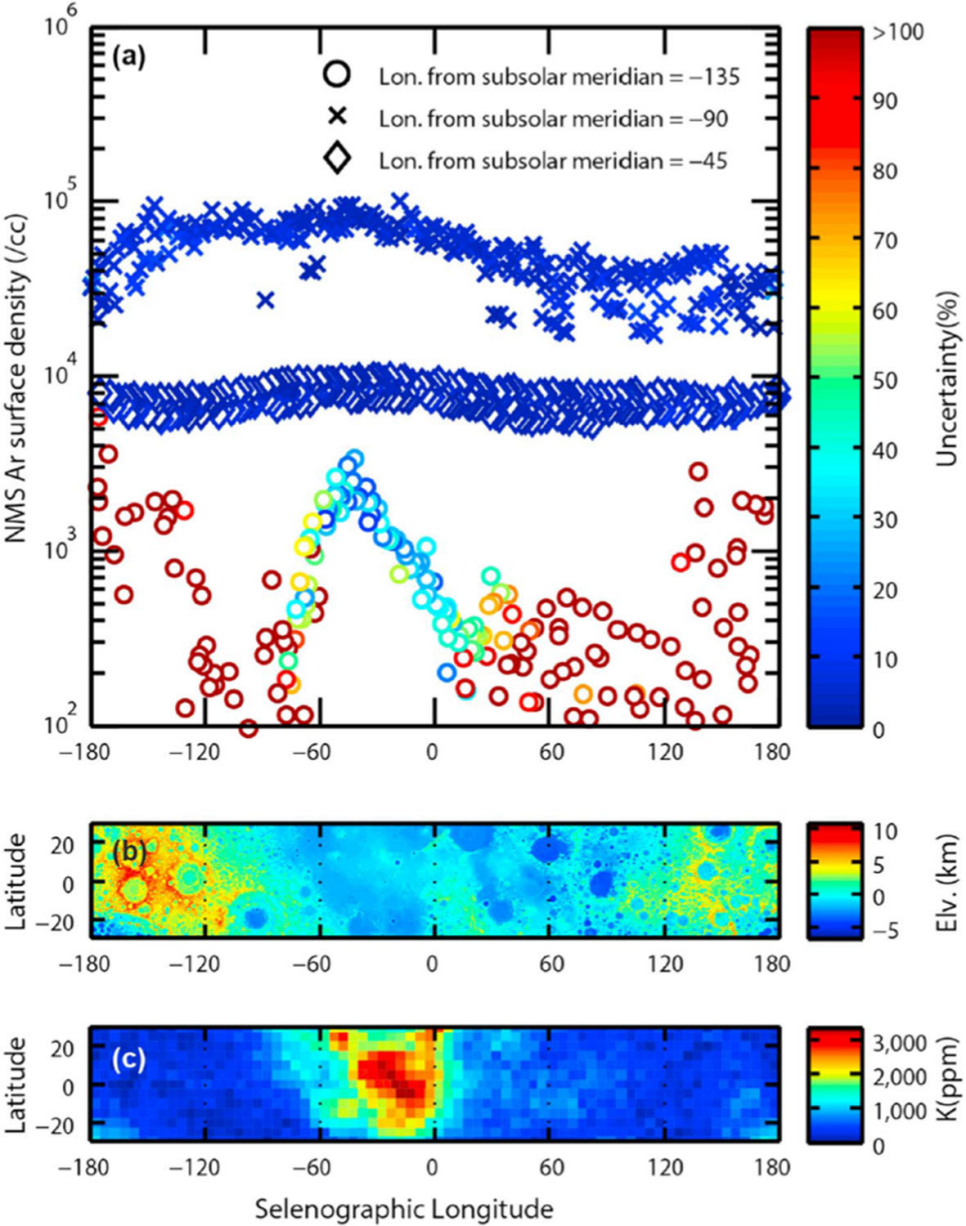
Exospheric densities of ^40^Ar measured at dawn (circles in top panel) are greatest above the western maria (middle panel), which are rich in KREEP elements, particularly ^40^K (bottom panel), which is the radioactive parent of ^40^Ar. Reproduced from Benna et al. (2015)

This kind of behavior was not expected from a noble gas. The exospheric model that best reproduced LACE observations required a heat of adsorption $Q$ for ^40^Ar on the lunar surface of ∼6500 cal mol^−1^, much higher than the value derived by adsorption experiments of ^40^Ar on glass (∼3800 cal mol^−1^; Clausing 1930). The heat of adsorption factors into the equation for the residence time of argon-40 atoms in a grain:

$$ t_{\mathit{res}} = \frac{C}{T^{2}} \exp \left ( \frac{4.19\boldsymbol{\cdot }Q}{\mathit{RT}} \right ) $$

where $Q$ is the heat of adsorption, $C$ is a constant (expressed in s K^−2^), $R$ is the gas constant, $T$ is the surface temperature (in K), and 4.19 is the conversion factor between calories and Joules. Hodges (1980) attributed this very high value of $Q$ for argon-40 (compared to laboratory measurements) to the high cleanliness of soil grains, which have been exposed for billions of years to the solar wind. Because it sticks efficiently to the cold lunar surface, ^40^Ar can be trapped in Permanently Shaded Regions (PSRs), areas at the lunar poles that never receive direct sunlight. The facts that argon is an endogenic gas, sticks at the surface, and can be deposited in PSRs where it can reside undisturbed for billions of years (Watson et al. 1961a, 1961b; Arnold 1979) make it a valuable species for studying the behavior of other molecules (most notably, water) that are difficult to measure (^40^Ar has been detected even at tens of km of altitude by LADEE/NMS). Grava et al. (2015) estimated that, during LACE measurements (∼9 months), 1,900 kg of ^40^Ar were deposited in PSRs poleward of 85° N/S, corresponding to 30% of the surface-ejected quantity, and that permanent cold trapping is a sink process for the exospheric ^40^Ar comparable in magnitude to photoionization and charge exchange with solar protons. Roughly four decades later, ^40^Ar was detected again in the lunar exosphere by LADEE/NMS, which confirmed the exospheric surface density but also revealed a bulge in exospheric density above Oceanus Procellarum (Benna et al. 2015; see also Fig. 5). This area (KREEP terrane) is rich in ^40^K, as measured by Lunar Prospector (Jolliff et al. 2000), and thus it is postulated that an enhanced diffusion of radiogenic gases occurs there. Two independent and concurrent simulations gave contradictory results, however. Hodges and Mahaffy (2016) found that the argon-40 bulge can be explained by a lower activation energy in that region and a very high activation energy (∼24,000 cal mol^−1^) everywhere else. On the other hand, Kegerreis et al. (2017) found that the bulge can be explained by an enhanced outgassing rate in that region (the western maria). Modeling LADEE/NMS data, they found that, in general, ^40^Ar has higher exospheric densities above maria, compared to highlands. This second explanation agrees with the hypothesis that circular fault systems around impact basins (with which the western maria are replete) are the regions where deep moonquakes are more likely to occur (Runcorn 1974).

Not all the argon atoms are readily desorbed at dawn. Some of them are temporarily sequestered at depth (where they arrived after diffusing downwards during the lunar night) and are released much later (mid-day). This mechanism, proposed by Kegerreis et al. (2017), could explain the slight time delay from dawn of the peak ^40^Ar exospheric density recorded by LACE and LADEE without requiring the high activation energy all over the lunar surface proposed by Hodges and Mahaffy (2016). Interestingly, a similar mechanism (the “thermal pump”) has been proposed for other species – most notably water – at the Moon (Schörghofer and Taylor 2007; Schörghofer and Aharonson 2014), Mercury (Vasavada et al. 1999), and Mars (Mellon and Jakosky 1993). It is therefore reasonable to expect that other species can behave the same way. Finally, the adsorbing behavior of ^40^Ar is such that it makes possible the creation of seasons. Data from LADEE/NMS were interpreted to be the result of seasonal migration of argon from one winter pole to the other (Hodges and Mahaffy 2016; Teolis et al. 2021).

**Fig. 6:**
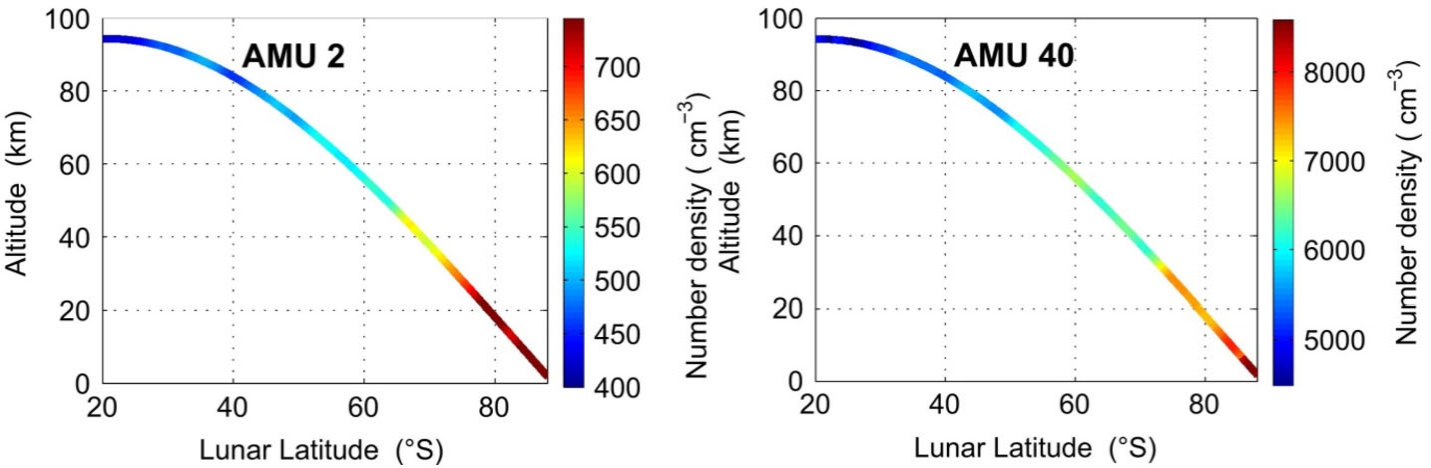
Number densities of H_2_ and ^40^Ar measured by CHACE onboard Chandrayaan-1 from the ∼100 km altitude above the subsolar point to the surface close to the poles. Reproduced from Thampi et al. (2015)

Argon was also detected by CHACE on its route to crash landing into a lunar south polar crater. Thampi et al. (2015) showed densities measured from 100 km altitude at 20° N latitude (∼5,000 cm^−3^) to ∼10 km altitude at the south pole (8,000 cm^−3^). This was the first detection in the polar regions (Fig. 6).

Argon has not been detected at Mercury. The Mariner 10 UVS placed only an upper limit of $6.6\times 10^{6}$ cm^−3^ (Shemansky 1988), from the difficult-to-observe emission doublet at 104.8 and 106.7 nm. The MErcury Surface, Space ENvironment, GEochemistry, and Ranging (MESSENGER) spacecraft (Solomon et al. 2007) did not carry a neutral mass spectrometer, and the bandpass of the primary exospheric instrument, the Mercury Atmospheric and Surface Composition Spectrometer (MASCS; McClintock and Lankton 2007) UV spectrograph did not include the wavelength of the ^40^Ar emission lines. In fact, the only way neutral argon-40 has been detected in exospheres so far is in situ mass spectrometry (LACE and LADEE/NMS at the Moon). Although Flynn (1998) claimed a detection of the argon doublet at the Moon from the ORPHEUS-SPAS II satellite, this detection was soon dismissed by Parker et al. (1998). Part of this spectroscopic non-detection can be explained by the low intrinsic brightness of the FUV ^40^Ar emission lines: Parker et al. (1998) found that these lines are optically thick (based on the densities retrieved by LACE), hence tens of times fainter than the HeI emission line observed by LAMP. Stern et al. (2012) note that these lines appear within detection capability of LAMP, but so far it has only placed an upper limit for ^40^Ar of $2.3 \times 10^{4}$ cm^−3^ (Cook et al. 2013). At Mercury, Killen (2002) estimated the column abundance of ^40^Ar of $5 \times 10^{8}$ – $2 \times 10^{9}$ cm^−2^ based on diffusion from anorthite in the top 25 km and a photoionization lifetime of 3.5 days at perihelion and 8 days at aphelion. This estimate of column density would make ^40^Ar one of the most abundant species in Mercury’s exosphere, but it is considerably lower than the estimated upper limit on argon column abundance of $5 \times 10^{12}$–$6 \times 10^{13}$ cm^−2^ from the UV spectrometer onboard Mariner 10 (Broadfoot et al. 1976).

At the Moon, LACE detected the less abundant isotope ^36^Ar, which is of solar wind origin. LACE showed a sunrise peak similar to ^40^Ar in time but 10 times lower in density: $3 \times 10^{3}$ cm^−3^ (Hoffman et al. 1973). This value of 10 for the ratio ^40^Ar/^36^Ar in the lunar exosphere is in contrast with the near equality of the two isotopes in returned soil samples (Table 3 in Yaniv and Heymann 1972). Therefore, the soil is not saturated with ^36^Ar, which means that the solar wind flux of ^36^Ar is permanently trapped. Excess of so-called “parentless” ^40^Ar in returned lunar samples, compared to expectations from solar wind composition and in situ decay of ^40^K, was suggested by Heymann and Yaniv (1970) to be of exospheric origin. This hypothesis was confirmed by Manka and Michel (1970), whose simulations showed that about 10% of the exospheric argon ions (^40^Ar^+^) are driven back towards the Moon instead of being entrained in the interplanetary magnetic field. These ions are then implanted into the lunar soil. Because these ions impact the lunar surface with energy of ∼1 keV, much lower than that of solar wind ^36^Ar ions (∼36 keV), they are not implanted as deeply as ^36^Ar^+^. Manka and Michel (1970) note that for this reason the ^40^Ar/^36^Ar ratio should vary with location: higher in surfaces parallel to the ecliptic plane (where mostly of these ^40^Ar^+^ ions impact); lower in surfaces facing the solar wind (which is rich in ^36^Ar). The ratio ^40^Ar/^36^Ar therefore offers the opportunity to study the amount of time a rock has been exposed to the surface and which orientation it had.

### Other Volatiles

Compared to the noble gases discussed above (argon and helium), far fewer observations exist of other volatile species. LACE made tentative detections of neon and methane, but those detections could barely be sifted out from contaminants. Recently, Killen et al. (2019) took advantage of the restoration of LACE neon data on NASA’s PDS archive and were able to model its behavior (Sect. 2.3.1). Methane was detected by LADEE/NMS, and Hodges (2016) showed that it can help understand the recycling of solar wind carbon at the Moon (Sect. 2.3.2). Hydrogen was detected at both Mercury and the Moon, but in different forms (molecular at the Moon, atomic at Mercury – see Sect. 2.3.3). Radon and polonium, two more species indicative of radioactivity in the interior of the Moon, were detected by the Apollo orbiters and by Lunar Prospector (Sect. 2.3.4). For several other species, LRO/LAMP provided more stringent upper limits for their lunar exospheric surface densities, most of them several orders of magnitude lower than previous estimates (Cook et al. 2013).

#### Neon

**Fig. 7:**
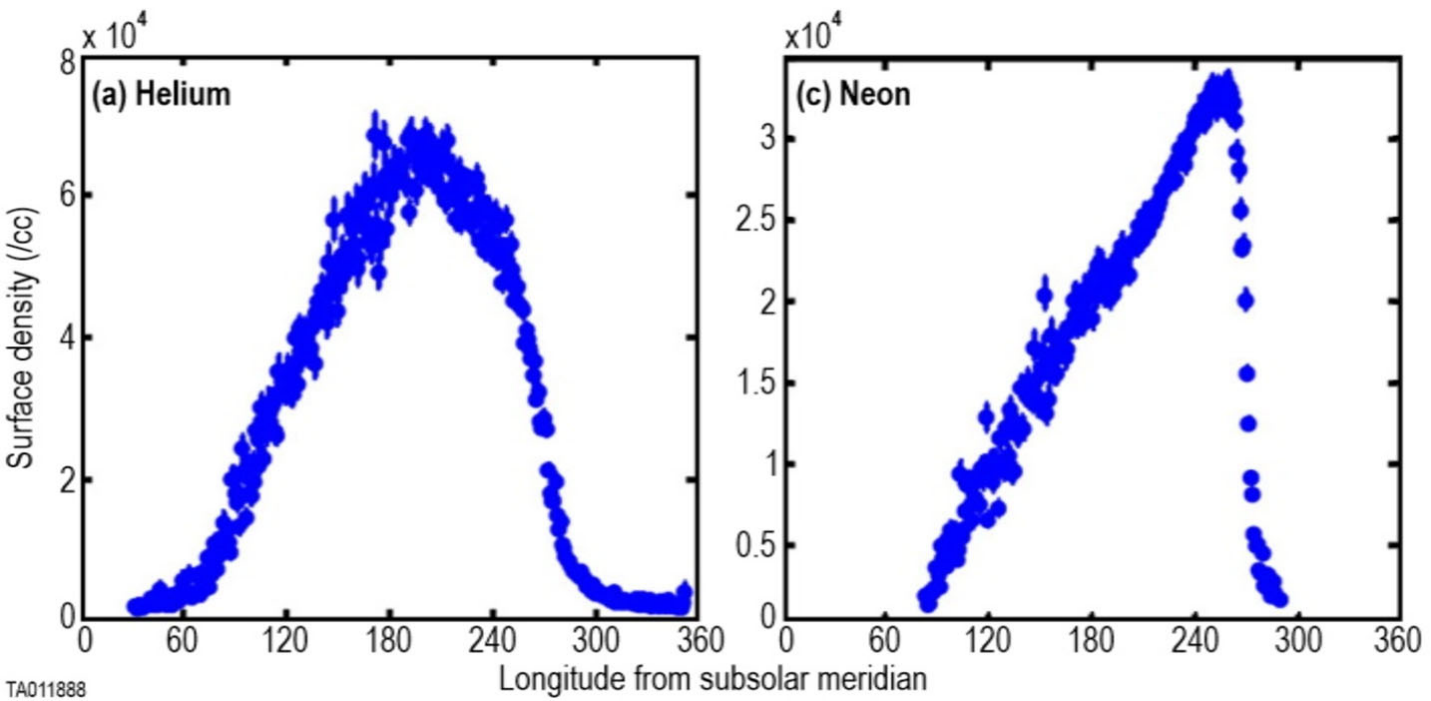
Surface densities for ^4^He (left) and ^20^Ne (right) inferred from LADEE/NMS measurements at altitude. These panels show the different behavior of these two species, mainly attributed to their different scale height. Adapted from Benna et al. (2015)

Neon (^20^Ne) was predicted to be the most abundant gas of solar wind origin in the lunar exosphere (Hinton and Taeusch 1964). Indeed, it was one of the first species indirectly detected in the lunar exosphere – as an ion – by the series of Suprathermal Ion Detector Experiment (SIDE) detectors deployed during the Apollo 12, 15, and 16 missions (Benson et al. 1975; Freeman and Benson 1977). Subsequently, it was detected in neutral form by LACE (Hoffman et al. 1973). These instruments reported surface densities of ∼10^5^ cm^−3^, confirming ^20^Ne as one of the most abundant species in the lunar exosphere. However, the ^20^Ne signature observed by LACE was attributed subsequently to $\mathrm{H}_{2}^{18}$O (Hodges et al. 1973), so these measurements were not considered further. Later, neon was measured by CHACE, the quadrupole mass spectrometer onboard Chandrayaan-1. The geometry of this spacecraft, en route to its impact point near the lunar South Pole, allowed it to measure neon in the dayside and over different ranges of latitudes. The number density reported varied from ∼2,000 cm^−3^ at the equator at 100 km altitude to ∼10,000 cm^−3^ at the poles close to the surface (Das et al. 2016). Subsequently (although results were published earlier), the LADEE/NMS also detected neon (Benna et al. 2015). During its 7-month long mission timeline, NMS reported neon densities slightly lower than those of helium, with peak density at dawn (2.0-3.5 × 10^4^ cm^−3^; see Fig. 7).

The NMS diurnal profile show a steady increase in ^20^Ne exospheric density from dusk to dawn, a sign of its non-condensable nature, but the exospheric density peak was recorded 10° (∼1 hour in local time) before dawn, instead of ∼2 AM local time in the case of helium. The difference in the two diurnal profiles is the result of the different scale height of the two species, and therefore of their different spatial extent. There is an inconsistency between ^20^Ne exospheric densities reported in the literature. The NMS surface densities (inferred from orbit) were an order of magnitude greater than the upper limits obtained remotely by LAMP from the emission line at 63.0 nm ($4.4 \times 10^{3}$ cm^−3^; Cook et al. 2013), but lower than those reported in situ by LACE ($1.1 \times 10^{5}$ cm^−3^; Hodges et al. 1974). Recently, LACE Ne data were restored, validated, and re-analyzed by Killen et al. (2019), which corrected the ^20^Ne measurement, considered to be contaminated by fluorine, using the ^22^Ne mass bin, supposed to be uncontaminated, and the known isotopic ratios of neon. This re-analysis reported much lower surface densities than those from Hodges et al. (1974): (1.5-4.5) × 10^3^ cm^−3^. One possible explanation of the discrepancy is that the value for Ne reported by Benna et al. (2015) was measured during a Coronal Mass Ejection (CME) passage (7-27 February 2014), which entails an enhancement in solar wind flux compared to the nominal conditions. If the lifetime of neon is the predicted 100 days for photoionization (Huebner and Mukherjee 2015), the exospheric density would be determined by the averaged solar wind influx during the previous three months. Simulations of the neon density using the photoionization lifetime of 100 days (and nominal solar wind conditions) reproduce LACE measurements, but are twice those from LADEE, taken during a CME. In order to reproduce the estimated surface density of Ne at the morning terminator of (2.0-4.5) × 10^3^ cm^−3^ by LAMP and (1.5-4.5) × 10^3^ cm^−3^ from the re-analyzed LACE data, a lifetime of 4.5 days is required (Killen et al. 2019). Furthermore, the reanalyzed LACE data indicate that the global diurnal distribution of Ne can vary over a lunar day, which is also consistent with a shorter lifetime than 100 days. The discrepancy between the data sets and the lifetimes is unresolved and requires further measurements.

At Mercury, Mariner 10 provided an upper limit for neon of $3 \times 10^{13}$ cm^−3^ (Broadfoot et al. 1974), from the 73.6 nm emission line. Because MESSENGER/MASCS did not have the capability of measuring the 73.6 nm line of Ne, there is currently no reliable measurement of Ne at Mercury.

#### Methane and Other Carbon-Bearing Species

**Fig. 8:**
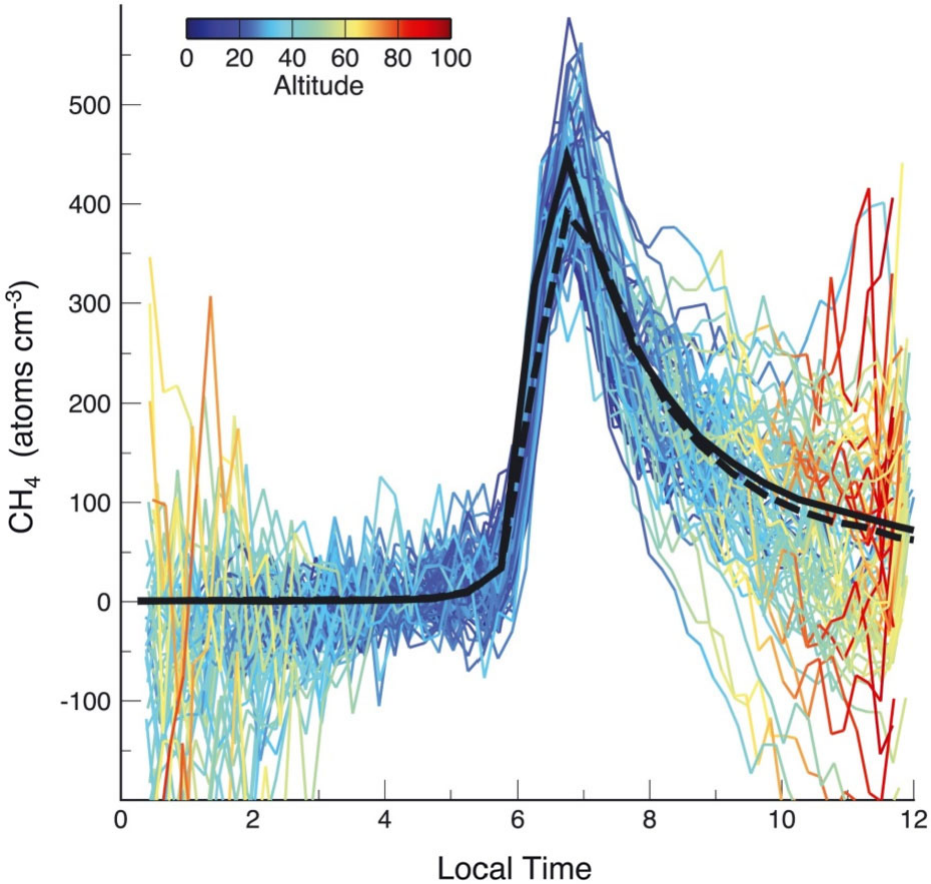
Methane number density measured by LADEE/NMS (colored lines) referenced to a common altitude of 12 km, around dawn. Black lines are exospheric simulations of methane. This figure shows the pronounced sunrise bulge in exospheric density, indicative of a species that condenses on the cold nighttime surface. Reproduced from Hodges (2016)

Methane (CH_4_) has been detected in the lunar exosphere by LADEE/NMS. Hodges (2016) reported observations taken close to the dawn terminator, where exospheric densities peak at a value of 400-450 cm^−3^ at 12 km altitude (see Fig. 8).

The diurnal profile reveals that CH_4_, like ^40^Ar, also adsorbs temporarily at the cold nighttime surface. However, the high activation energy (higher than that of argon) means that there is a delay of ∼1 hour in morning release (∼7 AM, instead of ∼6:30 AM for ^40^Ar).

**Fig. 9:**
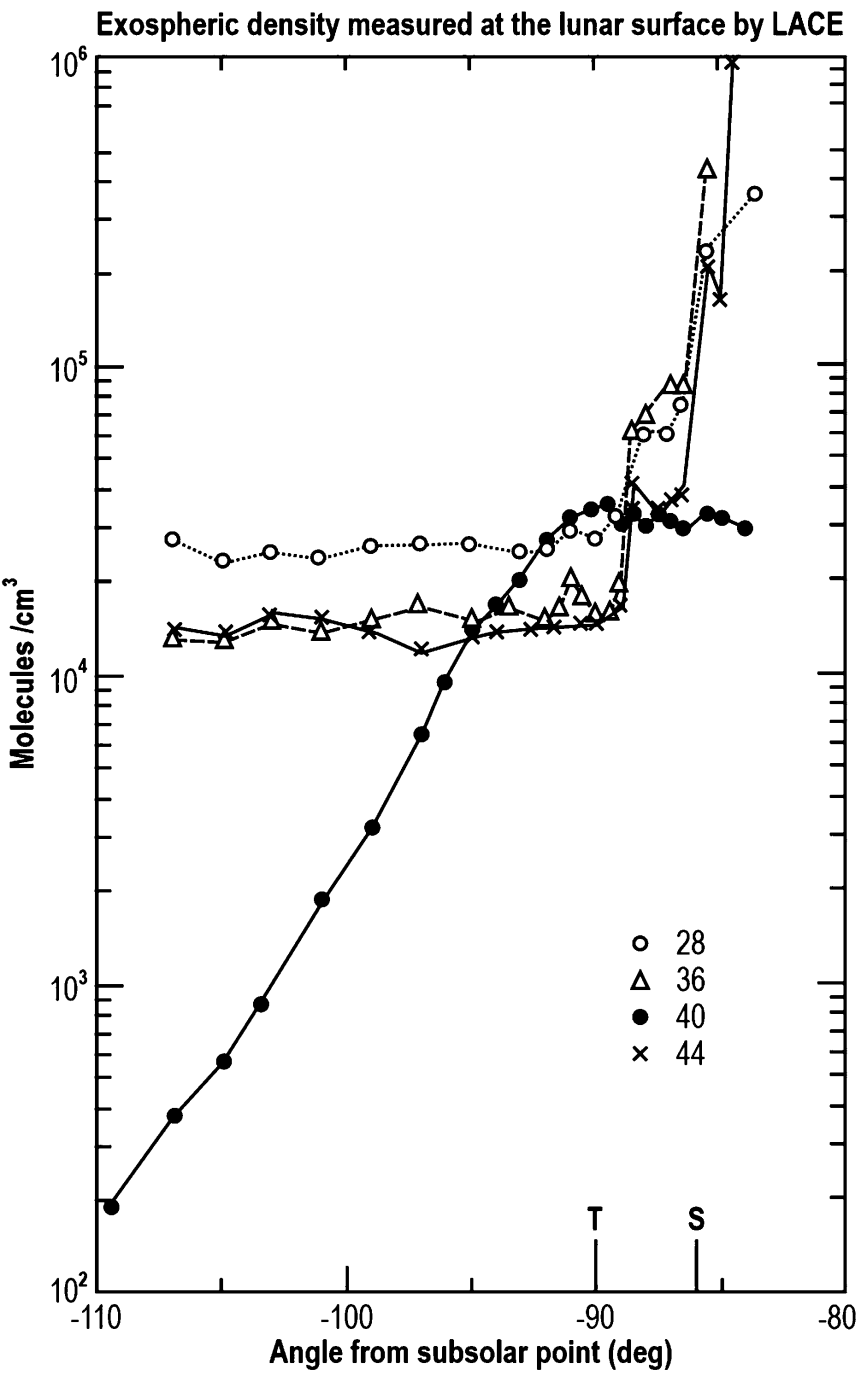
LACE exospheric density at the surface from four masses. Masses 40 and 36 are interpreted to be argon. Mass 28 could be N_2_ or CO. Mass 44 could be CO_2_. This species is expected to adsorb at the lunar surface, so the lack of such a bulge at dawn is surprising. T indicates terminator; S indicates sunrise (delayed from the terminator by ∼8 hours because of the mountains to the West of the Taurus-Littrow valley). Adapted from Hoffman et al. (1973)

Analysis of LADEE/NMS data (Hodges 2016) revealed that methane plays a role in the recycling of solar wind carbon nuclei impacting the lunar surface (as was suggested 40 years earlier by Hodges 1976), which then are lost from the exosphere owing to the low photoionization lifetime of CH_4_ (1 day). The delivery of solar wind C to the Moon is substantial: 8 tons/year (Hodges 1976). Because C abundance in returned samples (100 ppm, mostly in CH_4_, CO, and CO_2_) is less than the saturation level from the solar wind influx (200 ppm; Bibring et al. 1974) and is uniform over the maximum depth probed (250 cm), and because the reworking depth of the regolith owing to micrometeoroid gardening is just 10 cm in 10^9^ years (Gault et al. 1974; Costello et al. 2018), it was proposed that the carbon influx must be balanced by a substantial exospheric loss in molecular compounds, especially on the dayside (from the analogy with helium). The most probable candidates are CH_4_, CO, and CO_2_. These three species were not detected during the nighttime by LACE, most likely because of adsorption at the surface and low exospheric density (LACE minimum threshold was ∼100 cm^−3^; Hoffman et al. 1973). But around dawn LACE recorded peak concentrations at mass bins 28 (CO, but also possibly N_2_) and 44 (CO_2_) of 10^2^-10^3^ cm^−3^ close to dawn, with molecules coming from the hot dayside and traveling back towards the night (Hoffman and Hodges 1975; see also Fig. 9).

Hodges (2016) estimated the methane escape rate to be 1.5-4.5 × 10^21^ s^−1^, equivalent to 25-76% of the global carbon influx. This can be compared with solar carbon escape of $3.4 \times 10^{21}$ s^−1^, obtained separately by analyzing Apollo samples. This led Hodges (2016) to propose that “a significant fraction of C that enters the exosphere as methane escapes as CO”. In fact, exothermic reactions between solar wind C and the lunar soil would lead to the creation of CO, whose lifetime against photoionization is nine times that of CH_4_ and thus would constitute an even more substantial exosphere than methane itself. LADEE/NMS, which is about four orders of magnitude more sensitive to ions than neutrals, did not detect CO, but it detected CO^+^ (Halekas et al. 2015). The detection of CH_4_ and carbon ions (C^+^ and CO^+^), briefly discussed in Sect. 5.1, highlights the existence of a carbon cycle at the Moon.

Other species have been tentatively detected by LACE, as shown in Fig. 9. Mass 28 could be either N_2_ or CO. Neither of those adsorbs at equatorial cold nighttime surface temperatures, so no pre-dawn enhancement is expected. But CO_2_ (mass 44) does absorb at those temperatures, so it is surprising not to see the pre-dawn enhancement at mass 44 which is seen in ^40^Ar, another condensable species. From this lack of pre-dawn enhancement, Hoffman et al. (1973) estimated the dawn exospheric density of CO_2_ to be $3 \times 10^{3}$ cm^−3^.

#### Hydrogen

Given that ∼96% of the solar wind is composed mainly by protons, it was assumed that the Moon had a substantial dayside exosphere of hydrogen (at least 3 × 10^3^ cm^−3^, according to Hartle and Thomas 1974). It was therefore surprising that the Apollo 17 UVS spectrometer onboard the command module did not detect any hydrogen: Fastie et al. (1973) placed an upper limit for H (from the Lyman-alpha emission line at 121.6 nm) of 10 cm^−3^, and for H_2_ (from the Lyman and Werner bands in the FUV) of 1.2 × 10^4^ cm^−3^. Feldman and Morrison (1991) later revisited the UVS upper limit on H_2_ to be $9 \times 10^{3}$ cm^−3^. It was then speculated by Hodges (1973) that the reaction of solar wind protons with the lunar surface led to the formation of H_2_.

**Fig. 10:**
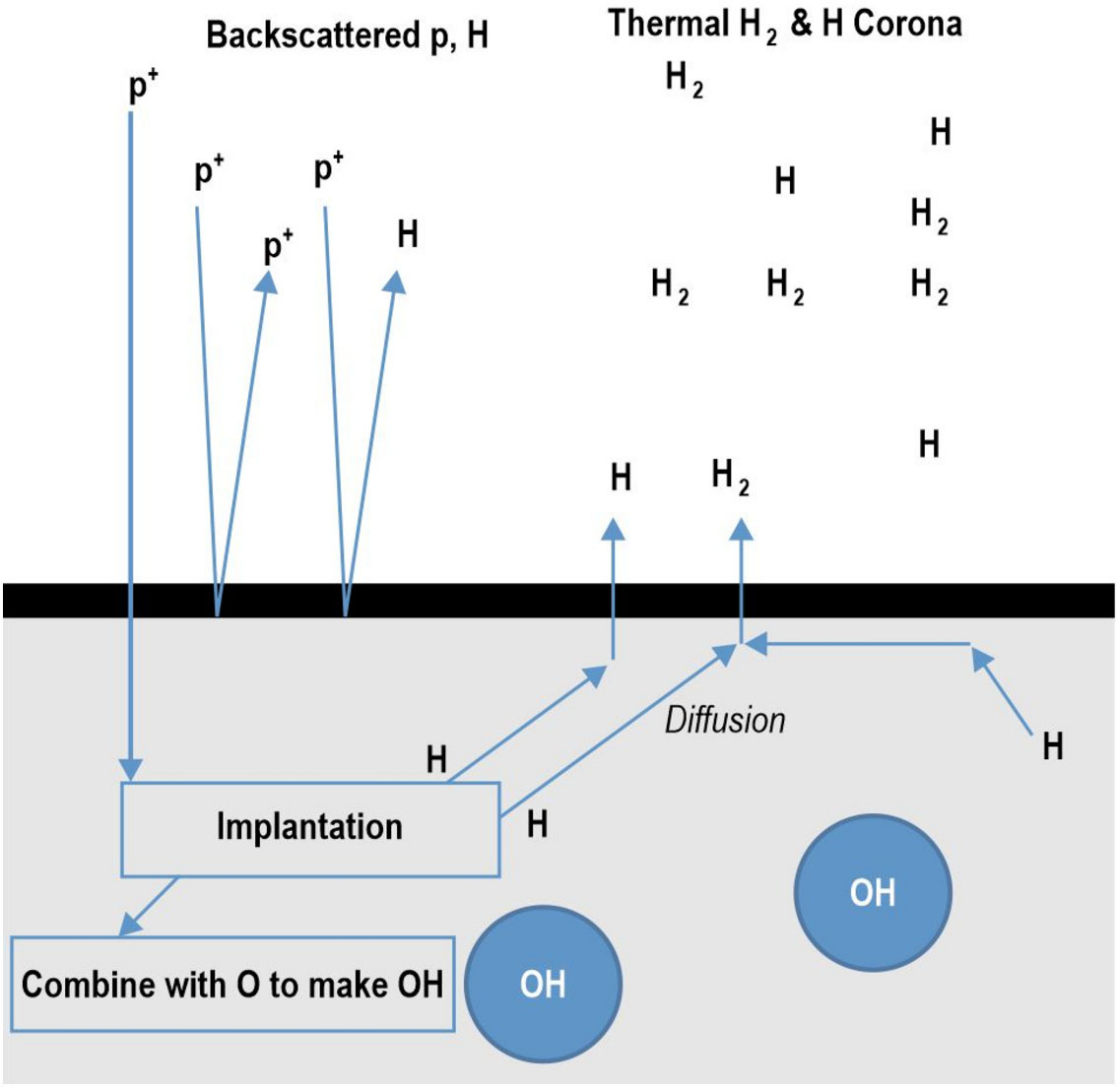
Mechanism (recombinative desorption) for the creation of H and H_2_ exospheres at the Moon or Mercury from solar wind protons and previously implanted H atoms. The diffusion rate depends on the temperature, whereas the implantation rate depends on the solar zenith angle. Reproduced from Tucker et al. (2019)

Molecular hydrogen is released into the exospheres of the Moon and Mercury by a process referred to as recombinative desorption (e.g. Starukhina 2006), which involves the diffusion to the surface of either bound H atoms released by chemical sputtering (Johnson and Baragiola 1991; Crider and Vondrak 2002), or freshly implanted H atoms (see Fig. 10).

The global content of H_2_ is balanced by the source of incoming solar wind protons, diffusion and formation of H_2_ in the surface, and the lifetime of H_2_ against thermal (Jeans) escape. The lifetime of H_2_ against photoionization (∼10^7^ s) is several orders of magnitude larger than thermal escape (Johnson 1971; Hodges 1974). Because H_2_ has a short thermal escape lifetime (hundreds of seconds for subsolar temperatures) compared to the orbital time of the Moon and Mercury, its global distribution is expected to vary directly with changes in the incident proton flux.

The discovery of widespread distribution of H_2_O/OH water on the lunar dayside by different instruments – Chandrayaan-1/M^3^ (Pieters et al. 2009), EPOXI/Deep Impact (Sunshine et al. 2009), Cassini/VIMS (Clark 2009), LRO/LAMP (Hendrix et al. 2019), and the SOFIA airborne telescope (Honniball et al. 2020) – has intensified the debate about the importance of the solar wind in the formation of lunar water (Schörghofer et al. 2021) through reactions between solar wind protons and oxygen (of which the lunar surface is replete). The Lunar Crater Observation and Sensing Satellite (LCROSS) experiment provided additional insight. Molecular hydrogen was detected among the species in the plume following the impact of the LRO Centaur rocket stage in the Permanently Shaded Region (PSR) of Cabeus crater. It was determined that the detected H_2_ was not the result of photodissociation of water, but was promptly formed by the impact via combination of two H atoms (Gladstone et al. 2010b; Hurley et al. 2012a). The discovery of energetic neutral hydrogen atoms and solar wind protons backscattered from the lunar surface (see Sect. 5) led Hodges (2011) to postulate that the majority of solar wind protons (98.5%) escapes the Moon as energetic neutral H, a negligible fraction (0.5%) is released as neutral H, and the remaining 1% is simply backscattered as ions. This work discarded the hypothesis that molecular hydrogen was an important constituent of the lunar exosphere. However, H_2_ was finally detected by LRO/LAMP on the Moon for the first time (Stern et al. 2013), from the Lyman and Werner bands. It took almost 4 years of twilight observations to build enough signal-to-noise: the spacecraft must be illuminated but the instrument must look at the dark lunar nightside to reduce the background; this geometry only occurs for a few minutes each orbit, near the poles and the terminator, except for when the spacecraft is orbiting along the terminator, but this geometry only occurs for a few days twice a year. The LAMP-derived global H_2_ surface density was 1200 ± 400 cm^−3^ (Stern et al. 2013). Modeling of LAMP observations by Hurley et al. (2017) showed that solar wind chemical sputtering is the dominant source of lunar exospheric H_2_, over micrometeoroid impacts and direct physical sputtering. Molecular hydrogen was also detected by the CHACE mass spectrometer onboard Chandrayaan-1, which provided the first detection of H_2_ on the dayside. The density was observed to vary in latitude, from ∼400 cm^−3^ at ∼100 km above the equator to ∼800 cm^−3^ at polar latitudes close to the surface (Thampi et al. 2015; see also Fig. 6). The lower densities probably reflect the fact that CHACE observations were carried out when the Moon was inside the geomagnetic tail, which shields the Moon from the solar wind. The LAMP observations showed a dawn/dusk asymmetry in surface density: 1,000 ± 500 cm^−3^ at dusk and 1,400 ± 500 cm^−3^ at dawn (Stern et al. 2013). This asymmetry was reproduced by the model of Tucker et al. (2019) which showed that the exospheric concentration of H_2_ is increasingly limited by H atom surface diffusion within the subsurface for activation energies > ∼0.52 eV. They showed that the variations, over a lunar day, of the rates of diffusion, which depends on temperature, and implantation, which depends on solar zenith angle, combine to give a slight increase of H_2_ near dawn compared to dusk. Moreover, using the averaged data of the solar wind flux incident on the surface in and out of the magnetotail, Tucker et al. (2021) showed that the H_2_ exospheric density decreases by an order of magnitude when in the magnetotail, a finding consistent with CHACE observations.

Considering the release of H_2_ from Mercury to be similar to the Moon, exospheric models have been used to estimate the global surface concentration (Killen and Ip 1999) and altitude profiles of density (Wurz and Lammer 2003). All models agree that H_2_ should be one of the most abundant species in Mercury’s exosphere, with surface densities on the order of 10^7^ cm^−3^. However, at the time of writing there are no published observational data of H_2_ in Mercury’s exosphere. Atomic hydrogen (H) has been detected at Mercury by Mariner 10’s UVS (Broadfoot et al. 1976) and MESSENGER/MASCS, thanks to the bright Lyman-alpha emission line (121.6 nm; McClintock et al. 2008). Mariner 10 observations revealed two populations, one “hot” at 420 K and one “cold” at 110 K. Work is in progress to model these two populations discovered by Mariner 10 and integrate them with MESSENGER observations, which show a morning enhancement in H above the dayside compared to the afternoon, as well as little emission from H on the nightside (Hurley et al. 2018). It is important to keep in mind that these Lyman-alpha observations are difficult to analyze owing to the substantial background, from both interplanetary hydrogen atoms resonantly scattering solar photons and from dayside scattering of solar H Lyman alpha photons.

#### Radon and Polonium

**Fig. 11:**
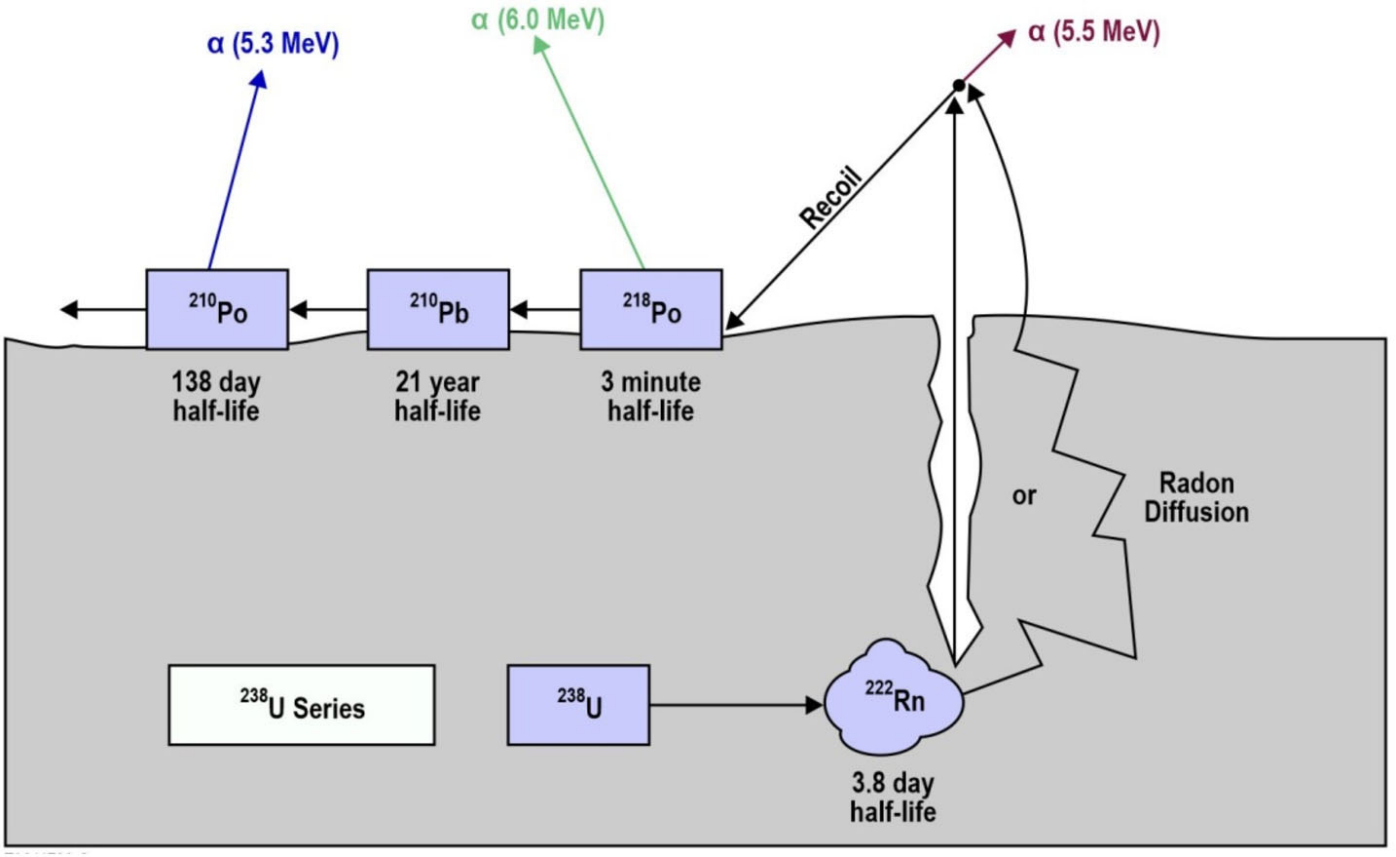
Scheme of radon decay, with alpha particle energies pertaining to each product. The short half-life of radon makes it a useful species to constrain regions of active outgassing. Adapted from Lawson et al. (2005)

Detections of alpha particles resulting from the decay of radon (^222^Rn) and its radioactive product polonium (^210^Po) were made by the alpha particle mass spectrometers onboard the Apollo 15 and 16 command module orbiters (Gorenstein and Bjorkholm 1973; Bjorkholm et al. 1973). Because radon is short-lived (half-life of 3.8 days), it represents another evidence that the Moon is actively outgassing radiogenic elements from its interior. Radon comes ultimately from the radioactive decay of ^238^U, and ^210^Po is one of its radiogenic daughters (see Fig. 11).

Because ^210^Po derives from ^222^Rn through the intermediate long-term decay of ^210^Pb, the two species constrain degassing over two different time scales: detection of alpha particles from radon indicates that the outgassing must have happened in the past few days, whereas detection of alpha particles from polonium indicates an outgassing that occurred decades earlier. Friesen and Adams (1976) showed that radon atoms don’t migrate directly from grains, where they are formed, to the void, but are carried by other radiogenic elements, for example ^4^He and ^40^Ar, during outgassing events. Such events may arise from tidal triggering of fault systems around maria (Runcorn 1977). Also, radon’s behavior after it is vented into the lunar exosphere mimics that of other condensable species, with ballistic random hops between one encounter with the surface and the next. The hop length is proportional to the temperature of the surface, so colder surface temperatures results in higher exospheric densities. If radon is vented into the cold nighttime surface, where the temperature is below its freezing point (211 K), it can be adsorbed until dawn, when it is promptly released similar to ^40^Ar (Heymann and Yaniv 1971; Lambert et al. 1977).

Enhancements of alpha particles from radon were detected above the edges of lunar maria (Gorenstein and Bjorkholm 1973), whereas enhancements of alpha particles from polonium were reported by the Apollo 16 alpha particle spectrometer near Grimaldi crater and the edge of Mare Fecunditatis (Bjorkholm et al. 1973). In a subsequent reanalysis of both spectrometers, Gorenstein et al. (1974) found enhancements of ^210^Po over edges of all observed maria except Serenitatis.

Other measurements of alpha particles were made by the Alpha Particle Spectrometer (APS) onboard Lunar Prospector (LP). When LP visited the Moon three decades after the Apollo measurements, it did not detect enhancements of ^210^Po alpha particles above some regions where detections were made by the Apollo orbiters, such as the Grimaldi crater (Lawson et al. 2005). LP/APS detected enhancements of ^210^Po alpha particles only above a few maria edges, in contrast with Apollo 15 and 16. One of the few regions that provided an enhancement of polonium in LP/APS data was the Mare Serenitatis, which in contrast was one of the few maria edges without a radon enhancement in the Apollo alpha particle spectrometer data (Gorenstein et al. 1974). This could mean that the radon release mechanism had abated from the Apollo era to LP measurements and/or that other regions have become (more) active (Lawson et al. 2005). Both the Apollo and LP alpha particle spectrometers reported radon release events at Aristarchus plateau (Gorenstein and Bjorkholm 1973; Lawson et al. 2005), which is rich in thorium and uranium. The Selenological and Engineering Explorer (SELENE; Sasaki et al. 2003) spacecraft also carried an alpha-ray detector (Nishimura et al. 2006), which reported enhancements in ^210^Po over Aristarchus, Imbrium, Serenitatis, and Moscovience maria despite instrument problems (Kinoshita et al. 2012).

## Refractories

Because of their much stronger bonds with the surface, refractory species are released into the exosphere by more energetic processes than the volatiles discussed earlier. Such processes include micrometeoroid impact vaporization (which peaks near dawn) and sputtering from solar wind and planetary ions. The escape processes for these species are also different. Whereas for light gases such as hydrogen and helium the gravitational (Jeans) escape dominates, photoionization and, to a lesser extent, charge exchange with solar wind ions (mostly protons) and electron impact ionization, are important loss mechanisms for refractories, even though a significant fraction of refractory species ejected by ion sputtering and impact vaporization has sufficient speed to directly escape. As for the volatiles, we concentrate here mostly on species that have been detected – all at Mercury (McClintock et al. 2018; Killen et al. 2018).

### Calcium

Calcium was first discovered in Mercury’s exosphere above the polar regions, through high-resolution observations from the Keck telescope of the emission line at 422.7 nm (Bida et al. 2000). MESSENGER/MASCS also observed the Ca emission line at 422.7 nm (McClintock et al. 2008). It was immediately recognized that the calcium in Mercury’s exosphere exhibited very high energies, with a scale height consistent with a temperature >20,000 K (Killen et al. 2005). Burger et al. (2012), using Monte Carlo simulations of the MASCS data, determined the Ca distribution was consistent with thermal temperatures of as much as 70,000 K (6 eV). Such high energies are necessary to loft the calcium to the high altitudes at which it is observed before it becomes ionized. This conclusion results from the very short photoionization lifetime of the calcium atoms, less than one hour at Mercury’s heliocentric distances (Huebner et al. 1992). Killen (2016) suggested that the large scale height of calcium must result from non-thermal processes. Specifically, that calcium is ejected from Mercury’s surface by impact vaporization in molecular form and subsequently dissociated by an energetic process such as photodissociation or electron-impact dissociation. The molecular compounds most likely involved are Ca(OH)_2_, CaOH, and/or CaO (Killen et al. 2005; Berezhnoy and Klumov 2008; Berezhnoy 2018). Using simple photolysis models, Berezhnoy (2013) estimated that the additional energy imparted to Ca-bearing products is 0.6 eV, <0.04 eV, and <0.6 eV for photolysis of CaO, CaOH, and Ca(OH)_2_, respectively. The photolysis steps are:

1. $\mbox{Ca(OH)}_{2} + \gamma = \mbox{CaOH} + \mbox{OH}$
2. $\mbox{CaOH} + \gamma = \mbox{CaO} + \mbox{H}$ or $\mbox{CaOH} + \gamma = \mbox{Ca} + \mbox{OH}$
3. $\mbox{CaO} + \gamma = \mbox{Ca} + \mbox{O}$

Therefore, it seems that even formation of Ca atoms via three steps of photolysis of Ca(OH)_2_, CaOH, and CaO is unable to produce Ca atoms hotter than about 1.2 eV (the sum of the three imparted energies). This is significantly lower than the 6 eV obtained by Burger et al. (2012). Another possible precursor molecule is CaS. Pfleger et al. (2015) have considered another process to generate energetic calcium: sputtering by solar wind ions precipitating at high latitudes through the magnetic cusps. They found that the Ca exospheric density produced by ion sputtering during nominal solar wind conditions can reach values of 1 cm^−3^, not insignificant when compared to the 1-4 cm^−3^ estimated by Burger et al. (2014). The density can reach even higher values than that if extreme solar events (like coronal mass ejections or high-speed streams) increase the area available to solar wind precipitating ions. Although considered to be a secondary process compared to impact vaporization and subsequent photodissociation, ion sputtering, which at Mercury predominantly occurs at high latitudes, can contribute to the calcium exosphere detected above Mercury’s poles by ground-based observations.

**Fig. 12:**
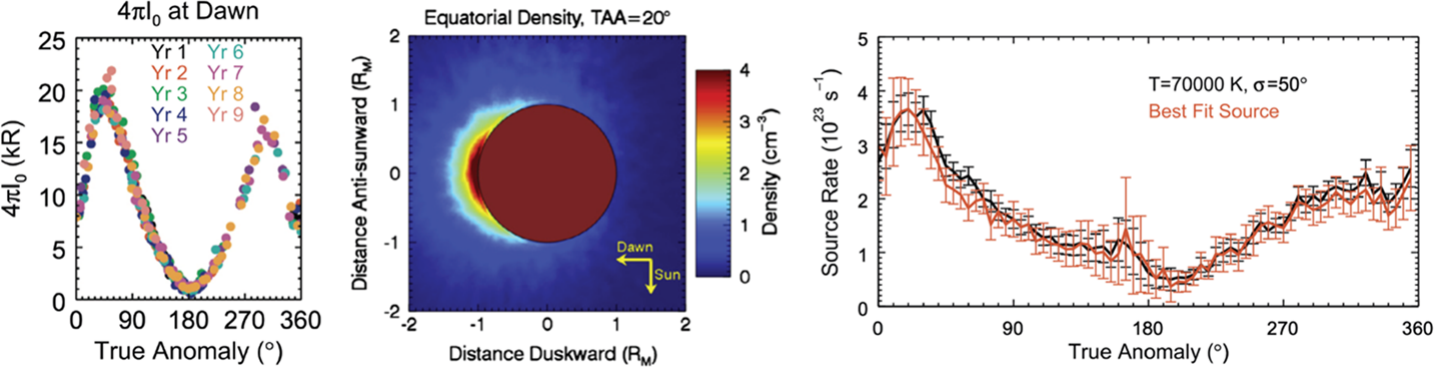
(Left) Intensity at the surface over Mercury dawn determined from exponential fits to MESSENGER/MASCS limb profiles. Different Mercury years are indicated by different colors. (Center) Ca density in Mercury’s equatorial plane at Mercury true anomaly = 20° based on the simple dawn-centered model of Burger et al. (2014) ($T = 70{,}000$ K, $\sigma = 50$°, source rate $= 3.7 \times 10^{23}$ s^−1^). (Right) Comparison of the source rate determined at all true anomalies using the simple model shown in the center panel to the best-fit source rate at each true anomaly. The simple model works remarkably well. Adapted from Burger et al. (2014)

The MESSENGER observations confirmed that Mercury’s calcium exosphere is centered on the dawn hemisphere and extends anti-sunward of the terminator, consistent with impact vaporization, which peaks at dawn (Pokorný et al. 2018) and indicating that the energization process is probably not photodissociation (Burger et al. 2012). Seasonal variations of the calcium exosphere were modeled by Burger et al. (2014) and subsequently used to determine that the calcium exosphere can be explained by an impact vaporization source centered at dawn. An excess of calcium near TAA = 20° was detected seasonally in the MESSENGER data and is likely due to the intersection of Mercury’s orbit with that of the comet 2P/Encke (Killen and Hahn 2015; TAA = True Anomaly Angle is Mercury’s angle, along its orbit, from perihelion). Further modeling of the comet 2P/Encke dust torus and its evolution under forces such as Poynting-Robertson drag confirmed the correlation between the position of the calcium excess and the comet Encke dust orbit relative to Mercury’s (Christou et al. 2015). Considering different exosphere generation and loss mechanisms, Plainaki et al. (2017) performed simulations of the Ca and CaO neutral environment using the 3-D Monte Carlo exospheric model of Mura et al. (2009). They found that the simulated morphology of the Ca exosphere is consistent with the available MESSENGER observations. According to Plainaki et al. (2017), the generation of a seasonal asymmetric CaO exosphere is expected, with the maximum surface release being on the dawnside-nightside hemisphere, near the equator, because there is where the comet stream particles preferentially impact the planet’s surface according to the model by Christou et al. (2015). In addition, an exospheric energetic Ca component, derived from the dissociative ionization and neutralization of CaO, is expected above the same region. The spatial distribution of the thermal Ca exosphere generated by photoionization of the CaO molecules in sunlight is expected to be asymmetric, exhibiting local maxima near the dawn region. Burger et al. (2014) found noticeable differences between the seasonal behavior of calcium and sodium. The Ca exosphere presents a fairly stable year-to-year seasonal dependence, with emission (density) peaks always occurring at dawn near the equator (see Fig. 12).

Thus far, no detection of exospheric calcium has been made at the Moon. The upper limit of the Ca column density in the lunar exosphere is estimated as $9.2\times 10^{7}$ cm^−2^ (Flynn and Stern 1996). It is possible to estimate the theoretical content of atoms of calcium (or other elements) in the exosphere using a stoichiometric model. A stricter upper limit of Ca column density, $5 \times 10^{7}$ cm^−2^, was obtained by Berezhnoy et al. (2014) with observations from the Zeiss telescope in Kabardino-Balkaria, Russia, and the Ca depletion factor relative to Na was estimated as >100. This limit is less than that expected from contributions by both impact vaporization and sputtering models (Sarantos et al. 2012). These observations can be explained by condensation of Ca-containing species in impact-produced clouds upon collisions between meteoroids and the Moon (Berezhnoy 2013).

### Magnesium

Magnesium (Mg) was discovered in Mercury’s exosphere from the emission line at 285.2 nm during MESSENGER’s second flyby (McClintock et al. 2009). Mg was found at high distances from the planet and high altitudes. Sarantos et al. (2011), analyzing the MASCS flyby data, found that the Mg exosphere is consistent with two populations: a hot component ($T > 20{,}000$ K) and a colder component ($T < 5{,}000$ K). MESSENGER orbital data analyzed by Merkel et al. (2017) showed that there is an enhancement in the exospheric Mg in the morning (6–9 AM local time) near perihelion, that the bulk temperature is ∼6,000 K, at times as low as ∼3,700 K or as high as ∼10,400 K, and that the production rate is strongest in the morning on the inbound leg of the orbit, i.e. TAA > 180°. Although Merkel et al. found occasional temperatures >10,000 K, consistent with the hotter component observed during the flybys (Sarantos et al. 2011), no observations from the orbital phase confirmed the colder component, although the lower end of the Merkel et al. temperatures (∼3700 K) is close to the upper end of the Sarantos et al. colder component (∼5,000 K).

**Fig. 13:**
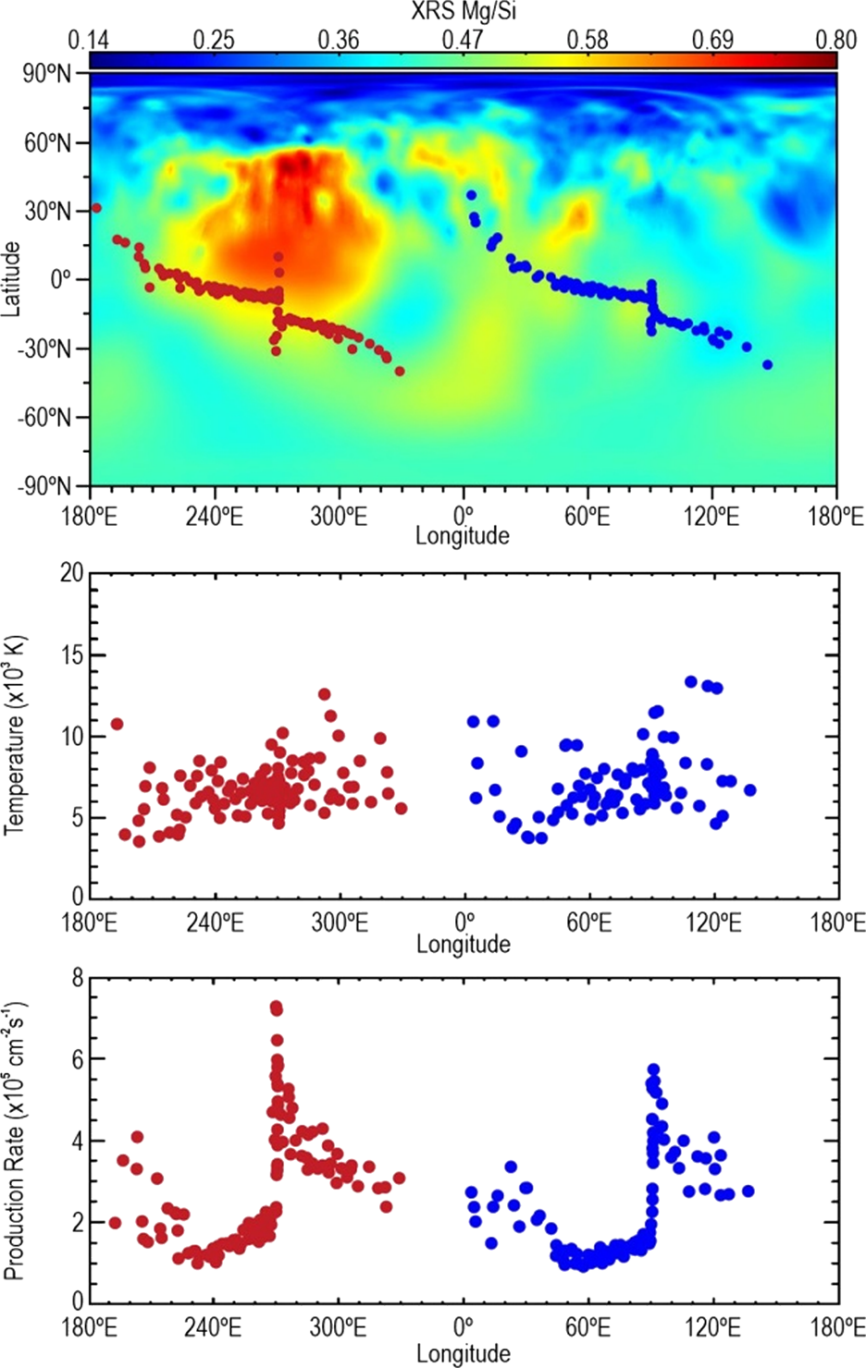
Summary of MESSENGER/MASCS observations of Mg over two Mercury years. Top: MASCS observations (circles, color coded by Mercury year) over a Mg/Si elemental weight ratio composite map derived from MESSENGER/XRS measurements (Weider et al. 2015). Middle: temperature fit (using the model of Chamberlain 1963) to MASCS observations. It shows how the temperature from the emission lines (4,000–8,000 K) is independent on the year. Bottom: the retrieved production rate of Mg. It shows how observations in red (years when the Mg-rich terrain is exposed at dawn at perihelion) are consistent with a higher production rate than observations in blue (years when the terrain antipodal to the Mg-rich terrain is exposed at dawn at the perihelion). Adapted from Merkel et al. (2018)

In a follow-up paper, Merkel et al. (2018) showed that the Mg column density is greatest over the Mg-rich terrain as measured by MESSENGER’s X-Ray spectrometer (XRS; Schlemm et al. 2007). Merkel et al. (2018) concluded that the main Mg source process is impact vaporization. However, the temperature as inferred from the scale height is almost twice that expected from impact vaporization. Figure 13 summarizes the Merkel et al. (2018) findings. Namely, the Mg source rate is higher for those years when the Mg-rich terrain is exposed at dawn at perihelion, compared to those years when the antipodal terrain is exposed at dawn at perihelion (because of the 3:2 spin-orbit resonance, a given longitude is exposed at a given local time every other year; Domingue et al. 2007). This is the first time that a direct link between the composition of Mercury’s surface and that of the exosphere has been established.

As with calcium, it is clear that at times an energetic process like ion sputtering or dissociation of a molecular precursor is responsible for ejection of Mg into the exosphere, but at other times impact vaporization dominates. Although the spatial distribution of Mg is not consistent with an ion-sputtering source, a portion of the atomic Mg could be from dissociation of a precursor molecule, similar to Ca. Quenching theory predicts that meteoroid bombardment is an effective source of MgO, Mg, and MgOH in the exosphere of Mercury (Berezhnoy 2018). The energy of Mg atoms produced via photolysis of MgO and MgOH is estimated as 0.4 eV and < 0.6 eV, respectively (Berezhnoy 2013). Agreement between observed and theoretical column density of Mg atoms from photolysis and impact vaporization (2 × 10^9^ cm^−2^; Merkel et al. 2018) suggests that meteoroid bombardment is the main source of Mg atoms in Mercury’s exosphere (Berezhnoy 2018).

There has been no detection of Mg in the lunar exosphere. The upper limit of the intensity of the MgI 285.2 nm emission line in the lunar exosphere was estimated as 53 Rayleighs, corresponding to an exospheric surface density of Mg of 6,000 cm^−3^, whereas the theoretical value from stoichiometric models is estimated as 476 R (Stern et al. 1997). LRO/LAMP placed an even stricter upper limit for the Mg surface density of 3.4 cm^−3^ near the terminator from the emission line at 182.8 nm (Cook et al. 2013). This value is slightly higher than that predicted by considering only sputtering as a source of Mg atoms in the lunar exosphere (1.0–1.5 cm^−3^; Wurz et al. 2007), whereas the expected near-surface density from impact vaporization was estimated to be 5 cm^−3^ (Sarantos et al. 2012). The difference between the stoichiometric model and observations can also be explained by less effective delivery of Mg atoms than Na atoms to the exosphere during meteoroid bombardment owing to condensation of Mg-containing species in collisions between meteoroids and the Moon (Berezhnoy 2013). However, it must be recognized that there is a substantial stoichiometric discrepancy between e.g. Na and O in Mercury’s exosphere. This discrepancy calls into question whether or not this is a viable assumption to estimate densities for certain species.

### Other Refractories (Al, Fe, Mn)

**Fig. 14:**
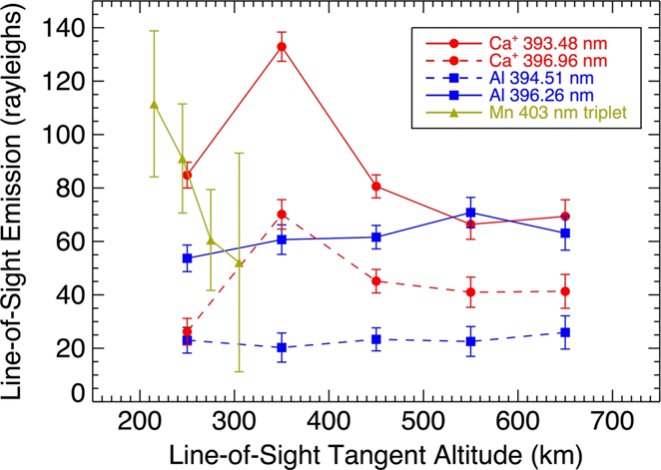
Line-of-sight tangent altitude profiles of Mn, Al, and Ca detected by MESSENGER/MASCS (spacecraft motion during the measurement of these profiles means they are not strictly radial profiles). The peculiar altitude profile of Mn, different from that of Ca^+^ or Al even though observed with similar geometry, when coupled with the timing in Mercury’s true anomaly angle, suggests that the Mn may be of cometary origin owing to a possible association with the comet 2P/Encke dust trail. Reproduced from Vervack et al. (2016)

A handful of other refractory species have been detected at Mercury by ground-based or MESSENGER observations. Aluminum (Al) and iron (Fe) were discovered using the Keck telescope (Bida and Killen 2011), and subsequently manganese (Mn) was discovered by MESSENGER/MASCS (Vervack et al. 2016). Whereas the Keck observations only detected a single line of Al, MESSENGER definitively confirmed the presence of the weaker ground-based detection by observing both lines of Al near 394-396 nm (Vervack et al. 2016). However, MESSENGER did not confirm the detection of Fe despite searches for several Fe lines. Al and Mn were only sporadically observed by MESSENGER, but there was a correlation between the TAA of the Encke-related peak in Ca and the TAA at which MESSENGER observed Al and Mn that suggests these two weakly emitting species may also be related to the comet Encke dust trail (Vervack et al. 2016). If this is the case, we might expect that the release of these species is dominated by meteoroid impact vaporization as with Ca, and that there might be an association, in part, with a molecular origin. Bida and Killen (2017) showed that Fe in Mercury’s exosphere increases with altitude, which is evidence for a molecular origin of the neutral atomic species, similar to Ca. On the other hand, in the ground-based observations, Al shows a more normal exponential decrease (Bida and Killen 2017), consistent with a hot exosphere (6,000–8,000 K) like that of Mg but not as extreme as that of Ca. Given that impact vaporization is expected to produce a plume at ∼3,500 K (e.g. Berezhnoy and Klumov 2008), some additional process is necessary to result in a >6,000 K exosphere. In contrast, the MESSENGER observations showed that Al may exhibit a flat to increasing profile with altitude, similar in structure to that found by Bida and Killen (2017) for Fe and thus suggesting a molecular species may be involved. MESSENGER observations of Mn show a completely different altitude distribution from that observed for Al and Ca^+^ (see Fig. 14). Given that the Al and Mn were observed at TAA roughly consistent with the comet Encke dust trail crossing, this different altitude structure may suggest a cometary origin for Mn, or at least a very different process for releasing Mn from Mercury’s surface (Vervack et al. 2016). However, both the ground-based and MESSENGER datasets probed the pre-dawn region of the exosphere where the effects of the planet’s shadow must be taken into account for the proper interpretation of any observations. Therefore, models need to be constructed to investigate the true profiles for all of these species.

Considering meteoroid bombardment as a source of Fe, Al, and Mn atoms in Mercury’s exosphere, the main Fe-, Al-, and Mn-containing species delivered to the exosphere via impacts are Fe, FeO, AlOH, AlO, Al(OH)_2_, and Mn (Berezhnoy 2018). The theoretical column density of impact-produced Fe atoms, $1.2 \times 10^{9}$ cm^−2^, agrees well with the observed column density ($8.2 \times 10^{8}$ cm^−2^; Bida and Killen 2017). However, photolysis of FeO leads to production of Fe atoms with energy of about 0.3 eV (Chestakov et al. 2005). This is significantly lower than the typical energy of Fe atoms observed in Mercury’s exosphere (∼1 eV; Bida and Killen 2017). This difference in energy of Fe atoms can be explained if Fe atoms are delivered to Mercury’s exosphere mainly by several steps of photolysis of impact-produced FeOH and Fe(OH)_2_ molecules and its photolysis products. The theoretical column density of photolysis-generated Al atoms, about $10^{6}$ cm^−2^ (Berezhnoy 2018), is significantly lower than the observed value, $7.7 \times 10^{7}$ cm^−2^ (Vervack et al. 2016). Such a low theoretical column density of Al atoms is explained by the effective condensation of Al-containing species during the expansion of impact-produced vapor. The theoretical column density of impact-produced Mn atoms during quiet times is about half the observed value, about $3 \times 10^{7}$ cm^−2^ (Berezhnoy 2018). This difference can be explained by an increased flux of impactors during the MESSENGER observations owing to timing of the observations and Mercury’s crossing of the comet Encke dust trail. It is expected that the initial temperature of impact-produced Mn atoms is about 3,000 K because Mn is produced mainly in the form of atoms during impact events (Berezhnoy 2018). However, the temperature of Mn atoms in Mercury’s exosphere has not yet been measured.

## Missing Species

There are several species that are expected to be present in the exospheres of the Moon and Mercury, some in quantities that should have been detected by the past or current instruments, but were not. On the Moon, these include for example nitrogen (N_2_), carbon dioxide (CO_2_), magnesium,, and calcium. The last two of these, plus mercury (Hg) and carbon monoxide (CO) were detected by LAMP in the LCROSS impact plume, as species permanently trapped within the Permanently Shadowed Region (PSR) of Cabeus crater and released by the impact (Gladstone et al. 2010b). For some of the other species, LRO/LAMP provided more stringent upper limits for the lunar exosphere, most of them several orders of magnitude lower than previous estimates (Cook et al. 2013).

Lithium (Li) is the third most abundant alkali element in the Solar System after Na and K. The average content of Na, K, and Li in norites in returned lunar samples is equal to 3,000, 1,500, and 12.3 ppm, respectively (Lodders and Fegley 1998). Lithium has a high emission rate (g-factor) for the 670.8 nm emission lines of 16 photons atom^−1^ s^−1^ at 1 AU (Sullivan and Hunten 1964; the g-factor $g$ is the number of solar photons resonantly scattered by each argon atom each second, and in optically thin exospheres it relates the observed intensity $I$ with the column density $N$ with the formula $I = g \cdot N$). This emission rate is higher than that of either the Na 589.0 nm or K 769.9 nm resonance lines, and thus it should favor the search for Li in the exospheres of the Moon and Mercury. However, Li has not been detected so far at either Mercury or the Moon. Several factors decrease the content of exospheric Li atoms. Its photoionization lifetime for quiet Sun, 5100 s, is much shorter than that of sodium (Na), $1.4 \times 10^{5}$ s, and potassium (K), $3.7 \times 10^{4}$ s (Huebner and Mukherjee 2015). Lithium is a light element, and as such it has a faster escape rate from the exosphere (especially at the Moon) in comparison with heavier Na and K atoms.

Spectroscopic searches for Li emission lines at 670.88 nm in the exosphere of Mercury were performed by Sprague et al. (1996) and by Doressoundiram et al. (2009), who reported upper limits for the zenith column density of Li atoms of $8.4 \times 10^{7}$ cm^−2^ and $4 \times 10^{7}$ cm^−2^, respectively. This column density can be compared to typical Na zenith column densities, $1.5 \times 10^{11}$ cm^−2^ (Potter and Morgan 1985) to give an upper limit for the Li/Na ratio on the order of $10^{-4}$. The Li content on the surface of Mercury is still unknown, so theoretical estimates of Li content in Mercury’s exosphere are absent. On the Moon, the upper limit of zenith column density of Li atoms in the exosphere is $1.1\times 10^{6}$ cm^−2^, from Flynn and Stern (1996). These authors also reported upper limits of intensities of resonance lines of other alkali atoms (230 Rayleighs for Rb at 780.0 nm and 520 Rayleighs for Cs at 852.1 nm), without converting them to zenith column densities owing to the lack of reliable g-factors (the unit Rayleigh is defined as: $1~\mbox{R}= 10^{6}/4\pi $ photons cm^−2^ s^−1^ sr^−1^; Hunten et al. 1956). The observations of Flynn and Stern (1996) were performed 20″ above the subsolar point near quarter Moon at the most suitable conditions to search for photon-desorbed exospheric atoms. The theoretical intensity of the Li emission lines at 670.8 nm in that region is estimated at 46 R, using a Li-Na stoichiometric model. The assumptions of this model are that the temperature of Na and Li atoms is the same (1,000 K) and that the physical parameters of Na and Li atoms in the exosphere and on the surface of the Moon (sticking coefficients, thermal evaporation rates, accommodation coefficients, diffusion coefficients) are the same. Differences in photoionization rates of Na and Li are also taken into account. However, the observed upper limit of the intensity of the Li 670.8 nm emission lines is only 17 R (Flynn and Stern 1996). Thus, one can tentatively conclude that the behavior of Li in the exosphere of the Moon is different from that of Na. An upper limit of Li zenith column density above the north pole of the Moon during the activity of the 2009 Perseid meteor shower is estimated as $4.9 \times 10^{6}$ cm^−2^ (Berezhnoy et al. 2014). The depletion factor of Li in the lunar exosphere in comparison with Na is found to be >1.6.

The behavior of Li during collisions of meteoroids with the surface of the Moon has been studied theoretically through quenching theory of the chemical composition of impact-produced vapor clouds. Impacts of meteoroids lead to delivery of LiOH, Li, LiO, and LiCl to the exosphere of the Moon (Berezhnoy 2013). LiOH is the main Li-containing impact-produced compound at temperatures of quenching of chemical reactions <3,700 K, typical for collisions of meteoroids exceeding 3 cm in radius. Photolysis lifetimes of LiO and LiCl at 1 AU for quiet Sun are equal to 28 and 225 s, respectively, whereas typical velocities of Li atoms produced upon LiO and LiCl photolysis are calculated as 2.6 and 3.8 km/s, respectively (Valiev et al. 2020). The LiOH photolysis lifetime at 1 AU for quiet Sun is estimated as 900 s, and the typical energy of Li atoms produced upon LiOH photolysis is estimated as 1.8 eV (Berezhnoy 2013). Therefore, photolysis lifetimes of the main Li-containing impact-produced species are shorter than or comparable to typical ballistic flight times of these species (${\sim} 10^{3}$ s). This leads to effective photolysis of impact-produced Li-containing species during the first ballistic flight and therefore to enhancement of hot photolysis-generated Li atoms in the exospheres of the Moon and Mercury during periods of active meteoroid bombardment. Such hot Li atoms could be detected during future observations of Li in the lunar exosphere.

Sulfur (S) is also expected to be present in Mercury’s exosphere, especially above the hollows and the Mg-rich areas, but it was not seen in the MESSENGER/MASCS spectra, most likely owing to its small g-factor. The sulfur surface abundance was published for some regions (Weider et al. 2015) and appears to be correlated with regions where Mg and Ca are also enhanced. Moreover, S is enhanced over its average abundance by up to a factor of 5 in the Mg-rich region (30°–60° N, 240°–300° E). In fact, it is speculated that the “light blue” regions surrounding the hollows are sulfur-containing volatiles (Nittler et al. 2011). Hollows are rare in the Caloris Basin (Thomas et al. 2014), where the surface concentration of S is also low (Weider et al. 2015). Theoretical estimates of the S column density in Mercury’s exosphere ($6 \times 10^{7}$ cm^−2^ from Wurz et al. 2010; $10^{9}$ cm^−2^ from Berezhnoy 2018; $2 \times 10^{10}$ cm^−2^ from Morgan and Killen 1997; and $2 \times 10^{13}$ cm^−2^ from Sprague et al. 1995) are inconsistent. Recent laboratory experiments suggest that photon-stimulated desorption of S from MgS, a proxy for the global form of S on Mercury’s surface, may provide a global, additional source of S at low altitudes of Mercury’s exosphere (Schaible et al. 2020).

Doressoundiram et al. (2009) reported upper limits for the Mercury’s exosphere of silicon (Si) of $5 \times 10^{10}$ cm^−2^) from the European Southern Observatory – New Technology Telescope in La Silla, Chile. An upper limit of Si from the Moon from Flynn and Stern (1996) appears to have been obtained using an excited line (390.6 nm) that is not expected to be populated (Sarantos et al. 2012).

Oxygen (O) represents a quandary. The published Mariner 10 results provide a generous upper limit for the O column density (emission line at 130.4 nm) of ∼10^11^ cm^−2^ (Broadfoot et al. 1974), on par with that of sodium. However, no oxygen emission at the 130.4 nm line (or the forbidden line at 135.6 nm) was detected with MESSENGER/MASCS, despite its higher sensitivity compared to the Mariner 10 UVS (Vervack et al. 2016). Column densities reported by Mariner 10 would have been detected by MASCS without difficulty. Vervack et al. (2016) proposed three explanations: the oxygen exosphere was significantly more abundant in 1974 than today; the Mariner 10 “detections” were only upper limits; or the Mariner 10 observations were somehow in error. On the Moon, oxygen has long eluded detection, both from mass spectrometers and from spectrographs. Hodges et al. (1974) noted that the absence of O and O_2_ in the lunar exosphere from the LACE mass spectrometer is understandable, if we consider that the Moon is less than fully oxidized, even though O is one of the major constituents of the lunar surface. LACE upper limits for molecular oxygen (O_2_) in the lunar exosphere were 100 cm^−3^ (Hoffman and Hodges 1975), which is roughly the sensitivity threshold of LACE (Hoffman et al. 1973). Oxygen has been detected on the Moon (Vorburger et al. 2014), but only as energetic sputtered species (see Sect. 5.2). The derived exospheric surface density (11 cm^−3^ at the subsolar point) is consistent with the LRO/LAMP upper limits (Cook et al. 2013) and predictions based on solar wind sputtering (Wurz et al. 2007).

A number of metallic constituents of the lunar exosphere were expected to be identified by the LADEE mission according to pre-flight calculations (Sarantos et al. 2012). Preliminary detections of Ti, Mg, and Al in the lunar exosphere were reported by Colaprete et al. (2016a) from the LADEE Ultraviolet/Visible Spectrometer (UVS; Colaprete et al. 2014). Line strengths of Ti and Mg decrease shortly after full moon, indicative of a dependence on solar wind. Line strengths of Al show a correlation with Geminids meteoroid stream, indicative of a meteoroid impact vaporization source. However, no density or column abundances have been derived to date from LADEE/UVS. The upper limit for exospheric surface density of Al from LRO/LAMP, 1.1 cm^−3^ (Cook et al. 2013), is close to the range predicted by considering sputtering as the main source of Al atoms in the lunar exosphere: 0.5–1.5 cm^−3^ (Wurz et al. 2007), but is lower than the density expected from impact vaporization (Sarantos et al. 2012). The efficiency of delivery of Al and Fe atoms to the lunar exosphere during meteoroid bombardment is not as high as that for alkali elements Li, Na, and K owing to condensation of Al- and Fe-containing species during expansion of impact-produced cloud and formation of slowly photolyzed Al-containing species in the impact vapor (Berezhnoy 2013).

## Ions and ENAs

Ions and Energetic Neutral Atoms (ENAs) are important to infer loss rates, interaction between the surface and the solar wind, and even properties of the neutral exospheres. We briefly summarize here the discoveries made on the Moon and Mercury. A more thorough analysis is reported in Wurz et al. (2021).

### Ions

Ions offer the opportunity to study the primary loss process of exospheric neutrals (with the exception of H and He, which escape predominantly with the Jeans mechanism), i.e. photo-ionization, electron-impact excitation, and charge-exchange with the solar wind ions (mainly protons). As Hartle and Killen (2006) have pointed out, with proper modeling tools it is possible to backtrace the ion to its origin at the surface, provided that the solar wind velocity and the interplanetary magnetic field are known. This is the technique used for example to infer exospheric properties from measurements of the lunar ionosphere (e.g. Poppe et al. 2013).

Ions of lunar origin have been measured on the surface by the SIDE detectors (Sect. 2.3.1), in lunar orbit by instruments onboard SELENE, Chang’E-1, LADEE, and ARTEMIS (e.g. Yokota et al. 2014; Saito et al. 2010a; Wang et al. 2011; Halekas et al. 2011, 2012, 2013, 2016; Poppe et al. 2012, 2016), and at more distant locations by instruments on board the WIND and AMPTE spacecraft (Mall et al. 1998; Hilchenbach et al. 1993). Detections or inferred detections to date include $\mathrm{H}_{2}^{+}$, He^+^, C^+^, O^+^, Ne^+^, Na^+^, Al^+^, $\mathrm{CO}^{+}$/$\mathrm{Si}^{+}$/$\mathrm{N}_{2}^{+}$, K^+^, Ar^+^/Ca^+^, and Fe^+^. The relative abundance of even the most common ion species remains in doubt, in part owing to the different observation geometries, but also to ambiguity regarding the source of the ions.

**Fig. 15:**
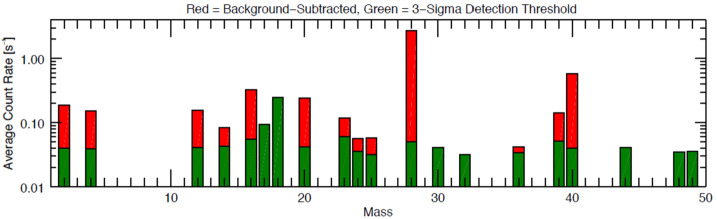
Mass spectrum of lunar ions detected by LADEE/NMS. Candidates for the substantial peak at m/q = 28 are $\mathrm{N}_{{2}}^{{+}}$, Si^+^, and CO^+^, with the latter one being the most plausible given the lower photo-ionization yields of the other two. Adapted from Halekas et al. (2015) with the addition of O^+^ signal at mass 16

Ions around the Moon come both from ionization of exospheric neutrals and directly from the surface (Yokota et al. 2009; Tanaka et al. 2009). The interactions of solar photons, solar wind ions, and interplanetary dust with the regolith can all lead to emission of both ions and neutral particles (Elphic et al. 1991; Madey et al. 1998). SELENE, Chandrayaan-1, and ARTEMIS detected low-energy protons reflected from the lunar surface. These measurements showed that between 0.1% and 1.0% of the incoming solar wind protons are backscattered (Saito et al. 2008; Lue et al. 2014, 2018). $\mathrm{H}_{2}^{+}$ was detected by LADEE/NMS (Halekas et al. 2015) and Solar Wind Ion Detectors (SWID) onboard Chang’E-1 (Wang et al. 2011). Recent analyses of SELENE data also reveal C^+^, apparently derived from the lunar surface (Yokota et al. 2020) and hinting at the importance of a carbon cycle at the Moon (see also Sect. 2.3.2). LADEE/NMS, which observed low-energy ions produced locally in the exosphere, found the highest fluxes (in order) for CO^+^/Si^+^/$\mathrm{N}_{2}^{+}$, Ar^+^/Ca^+^, O^+^, and Ne^+^ (Halekas et al. 2015; see Fig. 15).

The Ar^+^ and Ne^+^ signals appear consistent with neutral composition data that reveal high abundances of these noble gases (Benna et al. 2015). However, the peak at 28 amu remains puzzling, with CO^+^ the most plausible species (as noted in Sect. 2.3.2, neutral CO is difficult to measure owing to the instrumental background of LADEE/NMS). Neutral CO has not been identified in the lunar exosphere or in lunar polar deposits (where it could be released by micrometeoroid impacts or solar wind ion sputtering), but it is a byproduct of exothermic reactions involving solar wind C and the surface (Hodges 2016), and, as mentioned in Sect. 2.3.2, could represent a more substantial exosphere than CH_4_ (which peaks at a few hundreds of cm^−3^). Moreover, since CO can photodissociate to form O^+^ and C^+^, its presence may help explain the surprising detections of those two ions (also observed by other lunar missions), otherwise difficult to reconcile with spectroscopic limits of their neutral counterparts (Cook et al. 2013 and Sect. 4). SELENE detected O^+^ ions with energy 1-10 keV only when the Moon was in Earth’s plasma sheet: Terada et al. (2017) concluded that these are terrestrial oxygen ions transported to the Moon by Earth’s wind, reminiscent of the “shared” Earth-Moon neutral exosphere mentioned in Sect. 2.1.

**Fig. 16:**
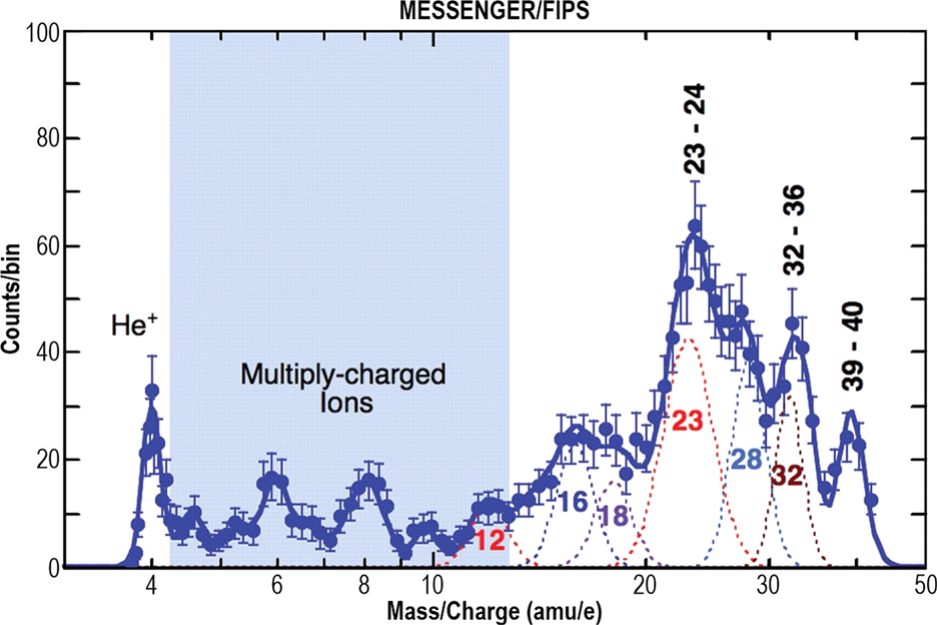
Mass spectrum of ions detected at Mercury by FIPS during MESSENGER’s first flyby (January 2008). Multiply charged ions (such as O^++^, Si^++^, and Mg^++^) are observed mostly below $\mbox{m/q} \sim 12$, even though Fe^++^ is observed at $\mbox{m/q} = 28$. Dashed curves are Gaussian fits to the major peaks, and the solid blue curve is their sum. Adapted from Zurbuchen et al. (2008). Reprinted with permission from AAAS

At Mercury, like on the Moon, ions of planetary origin come primarily from photoionization of exospheric neutrals and directly through surface processes (Killen et al. 2007). Most observations of Mercury planetary ions come from MESSENGER’s Fast Imaging Plasma Spectrometer (FIPS), part of the Energetic Particle and Plasma Spectrometer (EPPS; Andrews et al. 2007). MESSENGER reported He^+^, O^+^ and Na^+^ on essentially every one of the >4,100 orbits as well as in the initial flybys (Zurbuchen et al. 2008, 2011; see Fig. 16).

Two of these ions, O^+^ and Na^+^, are reported as part of mass per charge (m/q) groups, the O^+^ group (m/q 16-20) and the Na^+^ group (m/q 21-30), owing to the low resolution of the FIPS instrument. These ions are concentrated in several regions of Mercury’s magnetosphere, primarily the cusps and central plasma sheet (Raines et al. 2013). In the central plasma sheet, their density has been estimated at 0.1–1.0 cm^−3^ (Gershman et al. 2014), which is only about 10% of the H^+^ number density but up to 50% of the mass density there. Cusp densities have not been published but appear to be at least as high. One of the most surprising results from the first planetary ion measurements was the high energy of planetary ions in the northern magnetospheric cusp, with ions of energy >1 keV being regularly observed (Raines et al. 2014). That study also reported the first indications of ions upwelling in the cusp, possibly owing to solar wind sputtering there. MESSENGER observed planetary ions throughout the magnetosphere as well as in the magnetosheath and beyond the bow shock, though lower in numbers than the cusp or plasma sheet. Thermal ions were not observed directly (∼1 eV) as the lower energy bound of the MESSENGER instrument was about 50 eV (Andrews et al. 2007). Calcium ions (Ca^+^) have been detected by MESSENGER/MASCS (Vervack et al. 2010, 2016) through emission in the 393.5 and 397.0 nm lines but not with FIPS because of its low mass resolution and possible overlap with other ions such as K^+^. MASCS observed Ca^+^ emission in two instances. The first was during MESSENGER’s third flyby, when emission was observed in the region tailward of the near-planet reconnection line (x-line; Vervack et al. 2010). This implies that a convection mechanism in the magnetosphere may be at play. The similarity between Ca and Ca^+^ line of sight column densities for this observation was a surprise, because the two species have very different velocities (Ca^+^ 100 s of km/s; Ca: few km/s). The second instance was during the same observations in which MASCS detected Al and Mn (see Fig. 14), suggesting that there might be a connection to the enhanced neutral Ca abundances MESSENGER observed during the interaction of Mercury with comet Encke dust.

Despite Mercury’s planetary magnetic field, solar wind ions and electrons can still impinge on its surface, precipitating through the cusps, causing ion sputtering and electron-stimulated desorption (ESD). The behavior and effect of the solar wind precipitation has been modeled extensively (Kallio and Janhunen 2003a, 2003b; Massetti et al. 2007; Benna et al. 2010). It has been difficult to make a definite link between precipitation and exospheric production in observations, due at least in part to the dynamic nature of Mercury’s magnetosphere (Milillo et al. 2005), but several studies have provided indications of this connection. Orsini et al. (2018) showed that episodic enhancements in ground-based observations were associated with a passing Coronal Mass Ejection (CME). Jasinski et al. (2020) showed that short-term enhancements in Na-group ions outside Mercury’s bow shock could be most logically explained by an episodic and local enhancement in the Na exosphere. Raines et al. (2017) attributed a large but delayed increase in He^+^ to a several-day enhancement in the He exosphere, which in turn resulted from the impact of a CME particularly enriched in He^2+^, contrary to what was reported at the Moon, where the exospheric helium density measured by LADEE/NMS increased promptly with the passage of a CME (Hurley et al. 2016; see Fig. 2, where the passage of the CME is visible in the peak near day 400). Prior to MESSENGER, it was thought that the extreme solar wind environment at Mercury could lead to the stripping away of the entire dayside magnetosphere, causing direct bombardment by the solar wind across the full dayside surface (e.g. Slavin et al. 2007) like on the Moon. Following MESSENGER, it became clear that this was a much rarer condition, as Mercury’s planetary field would react via magnetic induction to counteract the effects (Slavin et al. 2014; Jia et al. 2019). However, a small number of “disappearing dayside magnetosphere” events were observed (Slavin et al. 2019; Winslow et al. 2020), where the closed field region of Mercury’s dayside magnetosphere was reduced below the altitude of the MESSENGER spacecraft (275–400 km). During these events, substantial portions of Mercury’s dayside surface may have been subjected to bombardment by solar wind plasma from Mercury’s magnetosheath. Sun et al. (2020), reported an analogous event on Mercury’s nightside, where the central plasma sheet may have been forced down to the nightside surface, from its normal position at hundreds km away.

### ENAs

**Fig. 17:**
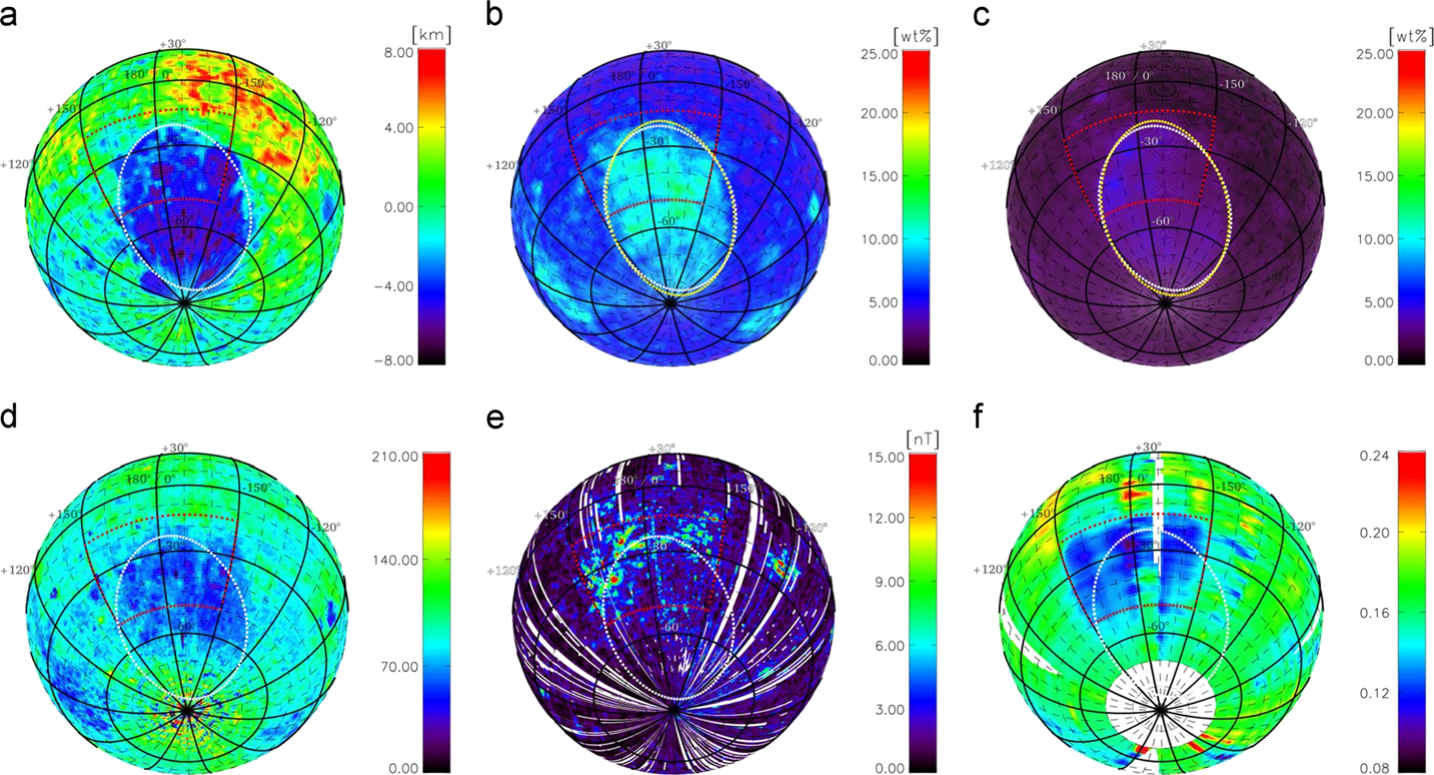
This composite image illustrates how ENA reflection (map in panel f) is predominantly correlated with lunar magnetic anomalies at the surface (see magnetic field at 30 km altitude from Lunar Prospector in panel e), rather than with topography (Clementine laser altimeter data in panel a), surface composition (Lunar Prospector gamma-ray spectrometer measurements of Fe and Th in panels b and c, respectively), or albedo (Clementine spectral reflectance mosaic at 750 nm in panel d). ENAs are therefore a useful tool for studying the exosphere-surface interaction, particularly on magnetic anomalies. Adapted from Vorburger et al. (2015)

Energetic Neutral Atoms (ENAs) are another useful tool to study the structure of the exosphere and its relationship with the surface. ENAs are solar wind ions that are backscattered as neutrals from the lunar surface with about 10% of the original particles’ energy. Traveling at about 140 km/s (∼100 eV) and being neutrals, ENAs travel in straight trajectories. Therefore, it is possible to trace detected ENAs back to their place of origin (much more easily than for ions) and to build a map of locations where ENAs are reflected (e.g. Vorburger et al. 2015; Lue et al. 2016; see Fig. 17).

With the ENAs mainly originating in the solar wind (see below for an exception example), most ENAs consist of hydrogen atoms. Interstellar Boundary Explorer (IBEX; McComas et al. 2009a) made the first detection of energetic neutral hydrogen at the Moon (McComas et al. 2009b). IBEX measurements were consistent with 10% of solar wind protons being converted in energetic neutral hydrogen atoms and reflected back with a broad energy range, in any case lower than the solar wind’s ∼1 keV. The Chandrayaan-1 Energetic Neutral Atom (CENA), part of the Sub-keV Atom Reflecting Analyzer (SARA; Barabash et al. 2009) onboard Chandrayaan-1, also detected energetic neutral hydrogen atoms, and the inferred fraction of solar wind protons reflected as such was higher: ∼20% (Wieser et al. 2009; Futaana et al. 2012).

Helium is the second most abundant element in the solar wind (∼3.8%), surpassed only by hydrogen (∼96%)—see e.g. Table 1 in Von Steiger et al. (2000). It is therefore expected that the total backscattered lunar ENA flux also includes reflected He particles. Indeed, in 2014, CENA measured for the first time alpha particles backscattered from the lunar surface as helium ENAs (Vorburger et al. 2014). The characteristic energy of the helium ENAs is roughly four times the characteristic energy of the hydrogen ENAs, agreeing with particle reflection theory. The measured helium to hydrogen ratio in the CENA mass spectra equaled $0.37 \times 10^{-3}$. Unfortunately, CENA’s geometric factor (detection efficiency) for helium has not been accurately determined, making it difficult to convert the measured He/H ratio into the He/H ratio actually present in the reflected ENAs. Based on experience with ENA instrumentation, though, the Vorburger et al. (2014) estimate that the actual He content is 10 times higher than determined, implying a He backscatter ratio of 1.4% (compared to the H reflection ratio of 16%).

**Table 1:** List of confirmed detections of neutral volatiles and refractories at the Moon and Mercury. We report here either surface number density (from in situ mass spectrometry) or column density (from spectroscopic observations). Values of densities in italic correspond to extrapolation at the surface from remote sensing or in-orbit mass spectrometer measurements, and they involve the convolution with an exospheric model

Species	Mercury	Moon	Reference
H	*8 cm*^−3^ *at subsolar point (thermal)* *80 cm*^−3^ *at subsolar point (non-thermal)*	–	Broadfoot et al. (1976)
H_2_	–	1.2 × 10^3^ *cm*^−3^	Stern et al. (2013)
He	4.5 × 10^3^ *cm*^−3^ *at the subsolar point*^∗^	(5-30) × 10^3^ cm^−3^ at dawn^+^	^∗^Broadfoot et al. (1976) ^+^Hoffman et al. (1973), Benna et al. (2015)
CH_4_	–	*450 cm*^−3^ *at dawn*	Hodges (2016)
Ne	–	(3-110) × 10^3^ cm^−3^ at dawn	Hodges et al. (1974) Benna et al. (2015) Killen et al. (2019)
Al	(1.9-7.7) × 10^7^ cm^−2^	–	Bida and Killen (2017) Vervack et al. (2016)
^36^Ar	–	4 × 10^3^ cm^−3^ at dawn	Hoffman et al. (1973)
^40^Ar	–	(2-10) × 10^4^ cm^−3^ at dawn	Hoffman et al. (1973) Benna et al. (2015)
Mn	4.9 × 10^7^ cm^−2^	–	Vervack et al. (2016)
Fe	8.2 × 10^8^ cm^−2^	–	Bida and Killen (2017)

Chandrayaan-1/CENA also measured lunar surface sputtered oxygen ENAs for the first time (Vorburger et al. 2014). These oxygen atoms do not originate in the solar wind, but are ejected from the topmost surface layer as the surface is irradiated with solar wind ions. Having characteristic energies of a few eV (compared to backscattered particles, which have characteristic energies of ∼100 eV; Wurz et al. 2021), these particles are on the lower end of the energy range covered by ENA detectors. Nevertheless, a clear, persistent oxygen signal was observed in the CENA mass spectra, amounting to ∼20-40% of the backscattered hydrogen ENA flux. Inferred surface and column densities were on the order of ∼10^7^ cm^−3^ and ∼10^13^ cm^−2^, respectively. The Advanced Small Analyzer for Neutrals (Wieser et al. 2020a) onboard the Yutu-2 rover of the Chang’E-4 mission also detected ENAs of mass larger than 4 amu at the lunar surface, highly variable in abundance and confined to energies below 100 eV (Wieser et al. 2020b). Whereas this is most probably also sputtered oxygen, the authors note that better statistics and more observations are needed for further characterization.

## Summary

We have discussed here species that represent the extrema of volatility (mobility) in surface-bounded exospheres in the inner Solar System. Each type of species adds a piece to the puzzle of the complex interaction between the airless bodies and the external environment (solar wind, meteoroids, and solar photons). Both the volatiles He and H_2_ shed light on the solar wind’s role in refilling the lunar exosphere, but each offers its own unique perspective on the exosphere production: H_2_ addresses the important aspect of what fraction of the lunar water is of solar wind origin; whereas He is a useful species to understand the still poorly known gas-surface interaction. Argon (^40^Ar), radon, and to a lesser extent helium, offer the tantalizing opportunity to quantify the amount of radiogenic elements of internal origin actively outgassing at present. On the other end of the range of mobility, refractories inform us of the importance of energetic processes (micrometeoroid impact vaporization and ion sputtering) in refilling the exosphere. Other papers that complement the topics discussed here are those on micrometeoroid impact vaporization (Janches et al. 2021), on particles and photons as drivers of exospheres (Wurz et al. 2021), and on surface-exosphere interaction (Teolis et al. 2021). Table 1 contains the list of species detected so far at the Moon or at Mercury.

We did not discuss asteroids. Although the presence of comae (and even of collisional atmospheres close to the nuclei) is well established for comets (and beyond the scope of this paper), for asteroids, which potentially represent the largest family of surface-bounded exospheres, the observations are still inconclusive. Morgan and Killen (1998) predicted that detection of coronae of two important species, Na and OH, around asteroids would be extremely challenging but not impossible from a spacecraft. There are active asteroids, also called “main-belt comets”, which spew dust grains when they are close to perihelion (see review by Jewitt 2012), and recently the Origins, Spectral Interpretation, Resource Identification, Security, Regolith Explorer (OSIRIS-Rex; Lauretta et al. 2017) has even detected cm-sized rocks being flung from asteroid Bennu (Lauretta et al. 2019), but there is a dearth of measurements regarding their exospheres. One exception is Ceres, for which there are detections of exospheric water-group species, including emission lines of hydroxyl (OH) at 309 nm (A’Hearn and Feldman 1992) and water (H_2_O) at 556.936 GHz (Küppers et al. 2014). However, the cases of non-detection of water-group species are just as numerous (Rousselot et al. 2011, 2019; Roth et al. 2016; Roth 2018). Right now there is no clear explanation for the origin of Ceres’ transient exosphere: sublimation rates from the known distribution of surface ice patches is two orders of magnitude lower than the water production rate derived from the observations (Landis et al. 2019). Villarreal et al. (2017) showed a correlation with solar energetic particle events, but ion sputtering is not effective enough (Küppers 2019).

The Rosetta mission, while en route to comet 67P/Churyumov-Gerasimenko, performed flybys of two asteroids: Steins (∼6 km size) and Lutetia (∼100 km size). Predictions of the exosphere of Steins and Lutetia were made by Schläppi et al. (2008) based on solar wind sputtering and impact vaporization, respectively. They predicted that a sputter-derived exosphere dominates over an impact vaporization-derived exosphere and that magnesium would be the dominant exospheric species after oxygen. They predicted that these detections would be challenging for the ion mass spectrometer ROSINA (Rosetta Orbiter Spectrometer for Ion and Neutral Analysis; Balsiger et al. 2007), but not impossible, at least at Lutetia. But ROSINA did not detect signs of their putative exospheres (Jäckel et al. 2010). Spacecraft outgassing, even after years of interplanetary travel, turned out to be a source of background gas contaminating the tenuous exospheric signal (Schläppi et al. 2010). ROSINA placed an upper limit for water of ∼3.5 × 10^3^ cm^−3^ from a closest approach distance of ∼3,000 km (Altwegg et al. 2012). Rosetta’s UV spectrograph Alice (Stern et al. 2007) also did not detect an exosphere around Lutetia (Stern et al. 2011).

## Future Steps

Despite the abundant progress made so far in the field of tenuous atmospheres, both observational and theoretical, several uncertainties still hamper our understanding of the inner Solar System exospheres and their interaction with the external drivers and the surface. For example, the column abundances that have been published so far using different observational techniques vary by orders of magnitude, and further observational, modelling, and laboratory advancements are needed. Here we briefly illustrate each of them.

### Remote and in Situ Measurements

The exospheres of Mercury and the Moon are notoriously difficult to study from the ground owing to several reasons: the extremely bright background from sunlight scattered from the surface and, in the case of Mercury, also the proximity to the Sun, which makes it visible for one hour at most during twilight. Nonetheless, ground-based observations have been for most of the time the only way to discover important exospheric species and to study how exospheres vary both in space and time due to variations in the external drivers. For example, observations of exospheric sodium (and to a lesser extent potassium) from ground-based telescopes have proven an essential tool to study source and loss process in the exosphere of both Mercury and the Moon (Potter and Morgan 1988, 1997; Tyler et al. 1988). Unfortunately, several of the species discussed here (Li, Mg, Ne, Ar) cannot be easily observed – or cannot be observed at all – from the ground. But for the few refractories that have been detected from the ground (Ca, Fe, Al), there is the need to perform additional observations to better understand their source and loss processes. For example, observations with adequate temporal coverage (observations over several consecutive nights) of calcium are needed to better constrain the dependence on the external drivers (micrometeoroid flux, solar energetic particle events, etc.). More precise line width measurements provide a more accurate temperature measurement of such gases.

At Mercury, the BepiColombo mission (Benkhoff et al. 2010), composed of two orbiters, the Mercury Planetary Orbiter (MPO) and the Mercury Magnetospheric Orbiter (MMO, also known as Mio), will provide a much anticipated comprehensive in situ study of its exosphere. In particular, the SERENA (Search for Exospheric Refilling and Emitted Natural Abundances) suite of instruments (Orsini et al. 2010, 2021) onboard MPO will make in situ measurements of neutrals and ions in Mercury’s environment. This suite of instruments will provide much needed constraints on the high-energy processes (micrometeoroid impact vaporization, ion sputtering) that refill Mercury’s exosphere. Spectra obtained by the PHEBUS (Probing of Hermean Exosphere By Ultraviolet Spectroscopy; Chassefière et al. 2010; Quémerais et al. 2020) ultraviolet spectrograph onboard MPO will be useful to supplement the mass spectrometer measurements for species (such as argon) ejected with low energy, and thus unable to reach the periapsis of 400 km of BepiColombo/MPO. PHEBUS large bandpass (55 to 315 nm) will allow it to detect several important species, like He, H, Mg. Complementing SERENA and PHEBUS, the Mercury Plasma Particle Experiment (Saito et al. 2010b) onboard Mio will directly measure charged particles in the exosphere and magnetosphere to quantitatively investigate generation mechanisms of the exosphere of each element. Thanks to the low-altitude orbit of MPO, regional and/or local time dependence of generation, escape, and circulation of heavy elements at the planet will be examined, together with observations from the Mercury Imaging X-ray Spectrometer (Fraser et al. 2010; Bunce et al. 2020) and the Mercury Gamma and Neutron Spectrometer (Mitrofanov et al. 2010), both onboard MPO. These instruments will measure the elemental surface composition of Si, Al, Fe, Mg, Ca, S, Ti, Cr, Mn, Na, K, P, Ni, U, Th, Cl, O, H and possibly C (Milillo et al. 2020). Finally, the Mercury Dust Monitor (Kobayashi et al. 2020) onboard MMO will provide measurements of dust impacts to the planet’s surface, much needed in order to constrain source processes of refractories. The synergy, unprecedented in Mercury exploration, of so many instruments in deriving important properties of the planet’s surface, exosphere, and magnetosphere will benefit future exospheric models (Milillo et al. 2020).

Regarding the Moon, great benefits would be achieved from orbiters, which would uncover temporal and spatial dependencies of exospheric abundances. This is especially critical in these times of renewed interest in lunar exploration. An assessment of the lunar exospheric composition is needed before it becomes forever changed: in a tenuous surface-bound exosphere like that on the Moon, every landing adds significant amounts of exogenic gases (Prem et al. 2020). As an example, each Apollo mission briefly doubled the mass of the lunar atmosphere (Vondrak 1974, 1992). Mass spectrometers can detect gases whose emission lines are too weak to be promptly detected by a spectrograph. For example, measuring the diurnal Ne abundance could resolve the discrepancy about its lifetime (Sect. 2.3.1). A measurement of ^40^Ar, coupled with the measurement of the ionized component (^40^Ar^+^), providing the loss rate for this element (photo-ionization and electron impact ionization being the major loss processes), would constrain the abundance of ^40^K within the crust and thus have important implications for the formation of the Moon (as well as that of Mercury). As LRO, SELENE, and LADEE have demonstrated, ultraviolet and visible spectrographs, especially onboard orbiters, also have proven useful to detect species over disparate locations and local times, uncovering temporal and spatial evolution of tenuous exospheres. There are plans to carry mass spectrometers on the lunar surface again, almost five decades since Apollo 17. Thanks to NASA’s Commercial Lunar Payload Services program, mass spectrometers (such as LEMS; Benna et al. 2020) will be deployed at the lunar surface. A network of mass spectrometers at different locations on the lunar surface will measure more gases than is possible from orbit, and will monitor their local time dependence (and thus their interaction with the lunar surface).

Regarding ions, published mass composition measurements made around the Moon display little consistency as to the ion species present or the relative abundance of different ions in the lunar exosphere. In part, this results from the wide range of ion mass composition measurement techniques utilized at the Moon, and the very different observational geometries employed by the various missions. In addition, there have been very few studies of the long term variability of ion composition around the Moon, which would provide a window on both the variability of the neutral exosphere and that of the ionization and transport mechanisms. Therefore, there is real value in performing ion composition measurements over a long duration, from a consistent observational platform. This science topic may be addressed at least in part by NASA’s HERMES (Heliophysics Environmental and Radiation Measurement Experiment Suite) and ESA’s ERSA (European Radiation Sensors Array) suites of plasma instruments planned to fly on the Lunar Gateway.

### Laboratory Measurements

As mentioned earlier, one of the biggest unknowns in the understanding of airless bodies’ exospheres is their interaction with the surface. To this regard, more laboratory experiments on gas-surface interaction are needed, for example studies on thermal desorption rates of argon and other adsorbers (e.g. Bernatowicz and Podosek 1991; Dohnálek et al. 2002; Patrick et al. 2015), necessary, for example, to refine the residence time of atoms on regolith grains. Also needed are experiments that refine the yields, cross sections, and threshold energy for photon-stimulated desorption (e.g. Schaible et al. 2020) and electron-stimulated desorption (e.g. McLain et al. 2011).

The dissociation cross sections of possible precursor molecules of Ca and Mg need to be measured or theoretically derived. These precursor molecules include CaO, CaOH, Ca(OH)_2_, CaS, MgO, MgOH, and MgS. These cross sections are particularly useful in understanding, for example, Mercury’s calcium exosphere and its extremely hot temperature (thousands of K). The energies distributions of the resultant atomic species should be derived. Rough estimates of photolysis lifetimes, as well as energy and velocity distributions of photolysis-generated metal atoms, need to be carried out using correlations between molecular properties of well-studied atmospheric species (e.g. Berezhnoy 2010). Complex modern *ab initio* models of photolysis currently have been applied only to diatomic molecules containing alkali metals (Valiev et al. 2020). Such models should be further developed for application to photolysis of polyatomic species including Ca, Mg, Al, and Fe. Moreover, a refinement of photoionization cross sections should be made for several atomic species, especially Ca, Ne, and Ar.

Finally, the renewed interest in the lunar exploration (like the NASA program Artemis) represents a compelling opportunity to bring back samples from previously unexplored regions of the Moon. For example, the ability to quantify the ^3^He and ^4^He content in new lunar samples would allow us to improve our knowledge regarding correlation between the ^3^He and ^4^He content and properties of the lunar regolith. This would lead to better constraints of ^3^He and ^4^He content on the surface of the Moon on a global scale.

### Simulations

Monte Carlo simulations of the lunar and Mercury’s exospheres are usually the best at reproducing the dependence of the exospheres from several parameters (solar radiation pressure, different source processes at the surface, ionization and charge-exchange on the dayside).

Recent works have illustrated the need for more accurate simulations of the surface-exosphere interaction in airless bodies. For example, Sarantos and Tsavachidis (2020) showed that the mobility of alkalis (Na and K) on the surface of regolith grains on both the Moon and Mercury reduce the overall desorption of these species from these grains. On the Moon, this effect might explain why the sodium exosphere reacts more slowly to the changes of the micrometeoroid flux compared to potassium, because the latter, being more massive than the former, has an overall reduced surface mobility, and therefore a higher chance to be photodesorbed. At Mercury this surface-diffusion dependence of photodesorption rate might explain the peak post-noon in the sodium exosphere at aphelion, which is not explained by models that assume that alkali atoms do not move on the grains. Such approach should be applied to other species, notably to another alkali element, lithium. Models should also include the temporary sequestration of adsorbed atoms in the subsurface. As discussed in Sect. 2.2, Kegerreis et al. (2017) showed that this process, by which argon atoms migrate downwards during the night and are released during the day later than dawn, can explain the half-an-hour delay in the sunrise exospheric density bulge measured by both LACE and LADEE.

Simulations of the lunar and Mercury’s exospheres should be run using the most-up-to-date information on the surface composition, including its surface variation (as was done e.g. in Colaprete et al. 2016b), using new information on the influx of micrometeorites and cometary material (Pokorný et al. 2018) and including their spatial and temporal variability (Pokorný et al. 2019).

Topography plays an important role in the transport of volatiles in airless bodies (e.g. Hodges 2011; Prem et al. 2018), and as such it should be included in exospheric models. It also affects the surface temperature. For example, it has been shown that the roughness of the lunar surface, casting both micro- and macro-shadows, affects the diurnal temperature profile, especially at the terminators, such that it deviates from a simple function of latitude and local time (Hurley et al. 2015). Therefore, one should include more accurate temperature maps, such as those from LRO’s Diviner radiometer (Williams et al. 2017). At Mercury, these maps will eventually be produced by the MERTIS radiometer onboard BepiColombo/MPO (Hiesinger et al. 2010, 2020) but there are already robust models for its surface temperature, validated by lunar parameters (Bauch et al. 2020).

Space weathering should also be included in exospheric modeling, to study how long ice frosts can reside in PSRs without being disturbed. For example, micrometeoroid bombardment, while being one of the source processes of surface-bounded exospheres, can also act as a loss process, especially for frost deposits in a PSR. This process also affects the lateral and vertical distribution of cold-trapped volatile deposits, as studies on micrometeoroid bombardment on water ice have shown (e.g. Crider and Vondrak 2003; Hurley et al. 2012b). Moreover, photo-destruction of adsorbed atoms or molecules by cosmic rays and Lyman-alpha photons from interplanetary hydrogen resonantly scattering sunlight should also be included (e.g. Morgan and Shemansky 1991).

New simulations should be done for ion sputtering loss rates using updated models of the ejecta angular and velocity distributions from experiments (like the SDTRimpSP code; Eckstein et al. 2007) and measurements of the solar wind and Mercury’s magnetosphere that will be provided by BepiColombo.

For Mercury, a reanalysis should be made of interplanetary dust and its spatial and temporal variability due to Mercury’s orbital parameters. This is especially true for the origin of refractories in Mercury’s exosphere. The existence and importance of nano-dust should be considered. Studies of the equilibrium condensation of dust particles were previously performed using limited thermochemical databases including mainly metal oxides. Adding silicate and non-silicate minerals to such thermochemical databases would allow us to study equilibrium condensation of species containing refractory elements during impact events in greater detail. Laboratory and theoretical studies of kinetics of formation of dust particles during impact events are also required for estimates of quenching parameters of condensation in impact-produced clouds.
